# Antibacterial Activity of PVA Hydrogels Embedding Oxide Nanostructures Sensitized by Noble Metals and Ruthenium Dye

**DOI:** 10.3390/gels9080650

**Published:** 2023-08-11

**Authors:** Diana Pelinescu, Mihai Anastasescu, Veronica Bratan, Valentin-Adrian Maraloiu, Catalin Negrila, Daiana Mitrea, Jose Calderon-Moreno, Silviu Preda, Ioana Catalina Gîfu, Adrian Stan, Robertina Ionescu, Ileana Stoica, Crina Anastasescu, Maria Zaharescu, Ioan Balint

**Affiliations:** 1 Faculty of Biology, Intrarea Portocalilor 1–3, Sector 5, 060101 Bucharest, Romania; diana.pelinescu@bio.unibuc.ro (D.P.); ileana.stoica@bio.unibuc.ro (I.S.); 2 “Ilie Murgulescu” Institute of Physical Chemistry of the Romanian Academy, 202 Spl. Independentei, 060021 Bucharest, Romania; manastasescu@icf.ro (M.A.); vbratan@icf.ro (V.B.); dmitrea@icf.ro (D.M.); mzaharescu@icf.ro (M.Z.); ibalint@icf.ro (I.B.); 3 National Institute of Materials Physics, 405A Atomistilor St., 077125 Magurele, Ilfov, Romania; maraloiu@infim.ro (V.-A.M.); catalin.negrila@infim.ro (C.N.); 4 National Institute for Research and Development in Chemistry and Petrochemistry-ICECHIM, 202 Spl. Independentei, 060021 Bucharest, Romania; catalina.gifu@icechim-pd.ro; 5 Techir Cosmetics SRL, Plantelor Str., 907015 Agigea, Romania; adrian.stan@yahoo.com

**Keywords:** optic active SiO_2_, TiO_2_, noble metal nanoparticles, sensitizer, singlet oxygen, hydroxyl radical, antibacterial activity

## Abstract

Nanostructured oxides (SiO_2_, TiO_2_) were synthesized using the sol–gel method and modified with noble metal nanoparticles (Pt, Au) and ruthenium dye to enhance light harvesting and promote the photogeneration of reactive oxygen species, namely singlet oxygen (^1^O_2_) and hydroxyl radical (•OH). The resulting nanostructures were embedded in a transparent polyvinyl alcohol (PVA) hydrogel. Morphological and structural characterization of the bare and modified oxides was performed using scanning electron microscopy (SEM), transmission electron microscopy (TEM), atomic force microscopy (AFM), UV–Vis spectroscopy, and X-ray photoelectron spectroscopy (XPS). Additionally, electrokinetic potential measurements were conducted. Crystallinity data and elemental analysis of the investigated systems were obtained through X-ray diffraction and X-ray fluorescence analyses, while the chemical state of the elements was determined using XPS. The engineered materials, both as simple powders and embedded in the hydrogel, were evaluated for their ability to generate reactive oxygen species (ROS) under visible and simulated solar light irradiation to establish a correlation with their antibacterial activity against *Staphylococcus aureus*. The generation of singlet oxygen (^1^O_2_) by the samples under visible light exposure can be of significant importance for their potential use in biomedical applications.

## 1. Introduction

In the last few decades, the global economic boom has triggered a tremendous search for better healthcare solutions and the preservation of quality of life, especially in densely populated communities where intensive natural resource usage has had an impact. One of the key challenges that emerged during this time was the increasing resistance of bacteria to antibiotics and the aggressive nature of cancer. Overcoming these challenges requires the validation of new antibacterial and theranostic agents.

Extensive research has been conducted to explore the interaction between inorganic materials, such as oxides and metals with endogenous compounds and living systems. The aim is to develop biocompatible nanostructures that can serve as safe implants or prosthetics and effective agents against various microbial strains. Among these materials, SiO_2_ and TiO_2_ have already demonstrated their biocompatibility and have been used as carriers for bioactive compounds such as drugs [1], enzymes [2,3,4], biological markers [5], and various implants [6,7]. However, to endow engineered SiO_2_ and TiO_2_ matrices with antimicrobial properties for biomedical applications, suitable synthesis conditions, modifiers/additives, and appropriate dispersion media for the powders are required.

In this regard, hydrogels based on polyvinyl alcohol (PVA) show promising properties as they can be used to embed oxide powders dedicated to the biomedical field. PVA is non-toxic, biocompatible [8], biodegradable [9,10], and transparent [11], making it an ideal candidate for such purposes. The transparency becomes particularly crucial when incorporating light-sensitive materials like TiO_2_, where its antibacterial properties are investigated not only in the dark but also upon exposure to light [12]. Furthermore, the incorporation of noble metal nanoparticles allows for the modulation of the antimicrobial reactivity of oxide matrices. Several studies have focused on metal nanoparticles and their effects on bacterial strains [13,14,15,16,17,18], providing valuable insights into antibacterial mechanisms [19], which are often modulated by surface plasmon resonance phenomena [20,21,22,23] when exposed to light.

The primary research focus of this work is the development of SiO_2_ and TiO_2_-based materials capable of displaying antimicrobial mechanisms both in the absence and presence of light. To accomplish this, the oxide matrices will undergo modification by incorporating noble metal nanoparticles such as Au, Pt, and ruthenizer, followed by their embedding in a PVA hydrogel.

Despite an abundance of information on microbial survival/extinction on engineered inorganic materials, predominantly metal, and metal oxide nanoparticles [24], a comprehensive understanding of reliable antimicrobial mechanisms, both with and without light irradiation, remains a challenge due to the varying responses of microbial strains to different environmental conditions. Generally, the action mechanisms of inorganic nanoparticles in the absence of light are evaluated based on the following factors: (a) ion release [25], (b) surface defects that mediate the generation of reactive oxygen species (ROS), and (c) bacterial cell damage [26]. When exposed to light, increased attention is given to photogenerated ROS, particularly oxygen singlet, which can serve as an antibacterial or theranostic agent [27]. For biomedical applications, including photodynamic therapy, various photosensitizers such as porphyrins [27,28], dispersed in different media like hydrogels [29], are employed to provide oxygen singlet. However, continuous efforts are being made to validate new organic compounds [30,31] for singlet oxygen generation, although many of these compounds are expensive and susceptible to physiological enzymatic activity. Therefore, developing more stable and affordable compounds that fulfill the desired functionality is crucial. 

One such material may be SiO_2_, which has primarily been used as a support for other active species without being recognized for its intrinsic activity [32]. Nonetheless, our previous studies on highly defected SiO_2_ with tubular morphology have revealed unexpected photocatalytic activity [33,34,35] and the ability to generate singlet oxygen under solar irradiation [35]. Herein, this activity was further investigated with visible light exposure and by using modifiers, as well as by comparing with TiO_2_-based samples. Cell viability of *S. aureus* has been monitored in the presence of modified SiO_2_ and TiO_2_ powders with gold, platinum, and ruthenium dye, before and after embedding in PVA hydrogel. The antibacterial tests were carried out both in darkness and under light exposure to examine the functional characteristics of light-sensitive materials (SiO_2_ and TiO_2_-based samples). Specifically, visible light irradiation and D_2_O were used to favor singlet oxygen generation, whereas the presence of hydroxyl radicals should be more pronounced in aqueous media under solar irradiation.

The aim of this study is to establish the effectiveness of bare and modified SiO_2_ and TiO_2_ powders, either in their standalone form or when incorporated into PVA hydrogels, as valuable antibacterial agents under dark and light exposure conditions. To achieve this, SiO_2_ nanotubes and TiO_2_ nanoparticles were synthesized using the sol–gel method, and further modified with ruthenium dye and noble metal nanoparticles (Au and Pt), with glutathione serving as a capping agent. The functional characterization of the synthesized samples was performed by monitoring the photogeneration of reactive oxygen species (hydroxyl radical and singlet oxygen) and conducting antibacterial assays against *S. aureus* in both dark and light-exposed conditions.

## 2. Results and Discussion

### 2.1. Electron Microscopy Characterization

**Figure 1:**
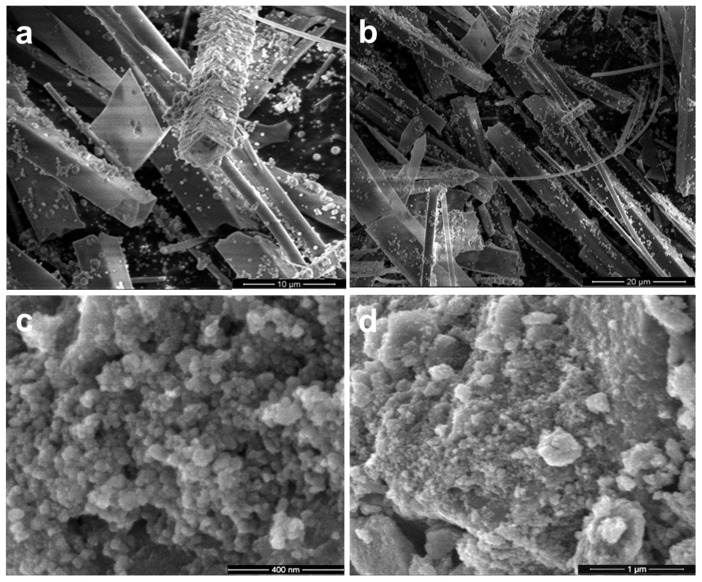
SEM images of bare SiO_2_ with tubular morphology (**a**)—scale bar 10 μm and (**b**)—scale bar 20 μm and, respectively, TiO_2_ nanoparticles (**c**)—scale bar 400 nm and (**d**)—scale bar 1 μm, before deposition of metal nanoparticles.

Figure 1a,b show SiO_2_ tubes reaching hundreds of micrometers in length and variable diameters from nano to micrometric range together with spherical particles located on their external surface, in line with our previously reported data [33,34,35]. Together with tubular morphology, the other structural and functional particularities (optical activity, singlet oxygen generation) of this atypical SiO_2_ are expected to be displayed. Figure 1c,d present the TiO_2_ nanoparticles about 20–40 nm, sticking together and developing big aggregates.

**Figure 2:**
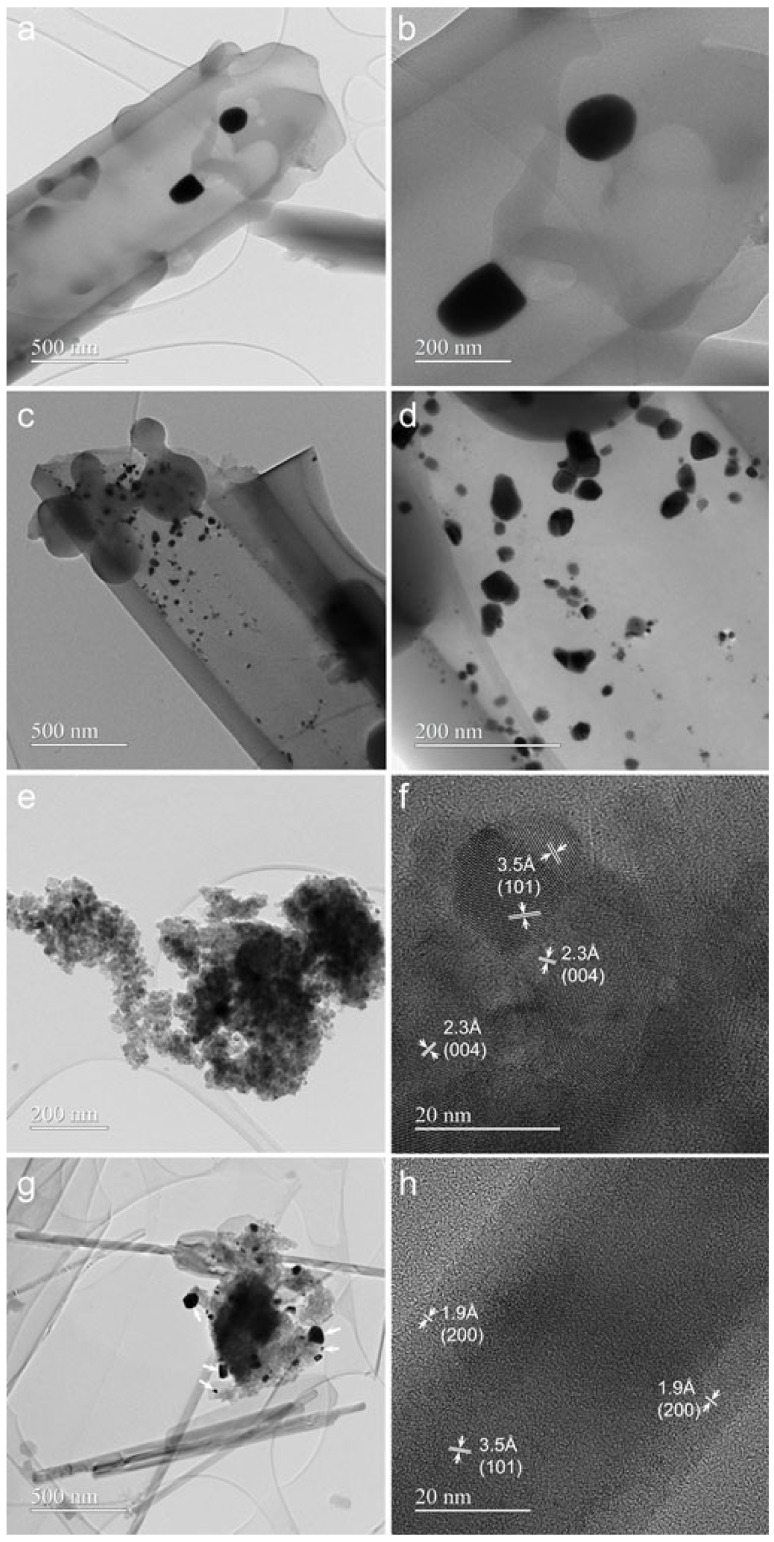
CTEM and HRTEM images of the SiO_2_ and TiO_2_ powder modified with metal nanoparticles: AuSiO_2_ (**a**)-scale bar 500 nm, (**b**)-scale bar 200 nm; PtSiO_2_ (**c**)-scale bar 500 nm, (**d**)-scale bar 200 nm; AuTiO_2_ (**e**)-scale bar 200 nm, (**f**)-scale bar 20 nm; PtTiO_2_ (**g**)-scale bar 500 nm, (**h**)-scale bar 20 nm.

Conventional TEM (CTEM) images (Figure 2) show the SiO_2_ with tubular morphology (Figure 2a) after modification with Au and Pt. The powders modified with Au (AuSiO_2_ sample) have lengths ranging from 2.3 to 15.7 μm and diameters from 110 nm to 820 nm. The size of Au nanoparticles, as illustrated in Figure 2b, varies from 60 nm to 120 nm. The SiO_2_ tube modified with Pt (PtSiO_2_ sample) shown in Figure 2c, has a length of 2.6 μm and a diameter of 0.9 μm. The size of Pt nanoparticles (Figure 2d) ranges from 4 nm to 63 nm. TEM also reveals that SiO_2_ tubes are amorphous in both samples.

In the case of Au-modified TiO_2_ material (AuTiO_2_ sample), only nanoparticles are present after TiO_2_ modification, as illustrated by the CTEM image (Figure 2e). The size of these nanoparticles varies from 7 nm to 29 nm. The high-resolution TEM (HRTEM) image (Figure 2f) demonstrates that the nanoparticles are well crystallized and characteristic anatase atomic planes are visible: 3.5Å corresponding to (101) planes, 2.3Å corresponding to (004) planes. The size of Au nanoparticles ranges from 80 nm to 210 nm. The CTEM image (Figure 2g) obtained for Pt-modified TiO_2_ material (PtTiO_2_ sample) shows two types of morphology: sheets and nanowires. The nanowires have lengths ranging from 0.6 μm to 2.7 μm and diameters from 20 nm to 108 nm. These are crystallized into an anatase structure as demonstrated by the HRTEM image (Figure 2h) where (101) and (200) planes are visible. The size of Pt nanoparticles (indicated by white arrows in Figure 2g) ranges from 17 nm to 93 nm.

### 2.2. AFM Characterization

**Figure 3:**
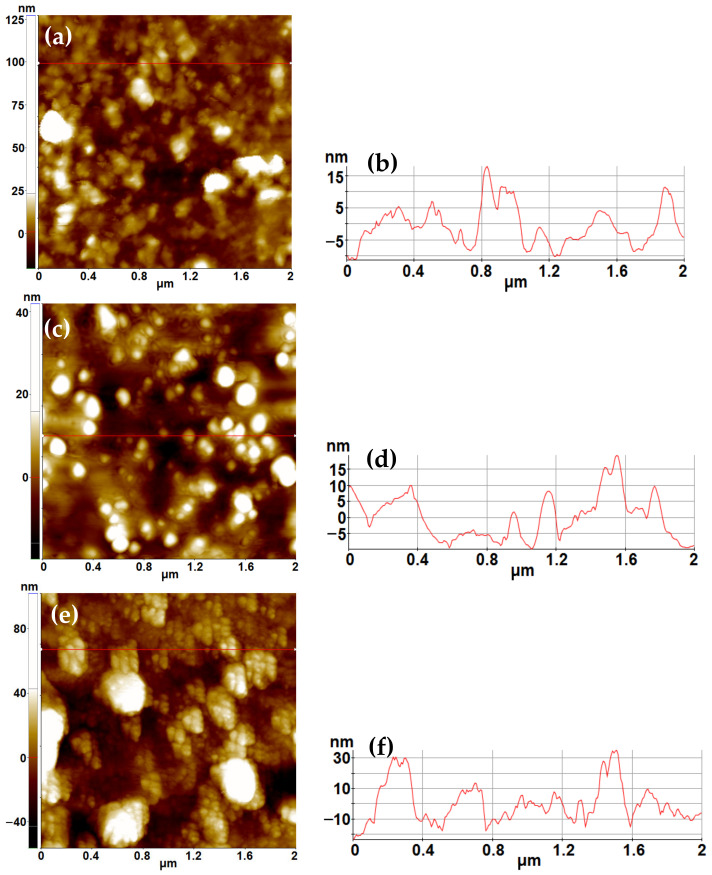
Topographic 2D AFM image, scanned over an area of (2 μm × 2 μm) of the prepared hydrogel, (**a**) together with a random height vs. distance plot (**b**); of the AuNPs (**c**) accompanied by a line-scan characteristic for the AuNPs (**d**); of the PtNPs (**e**) alongside with the corresponding profile (line-scan) of the PtNPs (**f**).

AFM investigations (Figure 3) were conducted to explore the morphology of the samples not characterized by other methods, namely: the hydrogel film and the free Au, Pt nanoparticles NPs (unsupported on SiO_2_ and TiO_2_ matrices).

Figure 3a presents the morphology of the prepared hydrogel, as seen in topographic images recorded by AFM at the scale of (2 μm × 2 μm). It can be observed that the surface of the hydrogel consists of a random agglomeration of material in the form of hills alternating with valleys (inter-hills free spaces), resulting in a height difference of a few tens of nanometers in the z-direction. For example, the line scan presented in Figure 3b suggests a height difference of approximately 25 nm (ranging from −10 to +15 nm). The entire scanned area shown in Figure 3a exhibits the following corrugation parameters: an RMS roughness (R_q_) of 11.6 nm and a peak-to-valley (R_pv_) of 147.4 nm. Figure 3c,e show the morphology of the Au and Pt nanoparticles. Both samples were prepared by their dispersion in ultra-pure water, followed by drop deposition on clean microscopic glass substrates. The AuNPs, exhibited in Figure 3c, have particles with diameters ranging from a few tens of nanometers, such as 30 nm, up to approximately 200 nm. However, most of the AuNPs shown in Figure 3c have a diameter of around 100 nm (consistent with the corresponding line scan plotted in Figure 3d. The PtNPs (Figure 3e) tend to cluster in bunches with cauliflower-like structures, with diameters in the range of 200–300 nm. The constituent particles within these bunches have a mean diameter of less than 50 nm (Figure 3e), also noticeable in the line profile from Figure 3f, indicating that each large particle (each peak) is composed of smaller neighboring particles, resulting in a “modulated” profile. The AFM characterization of Au and Pt-modified TiO_2_ and SiO_2_ powders is presented in Figures S1–S3 from Supplementary Materials.

### 2.3. XRD and XRF Characterization

**Figure 4:**
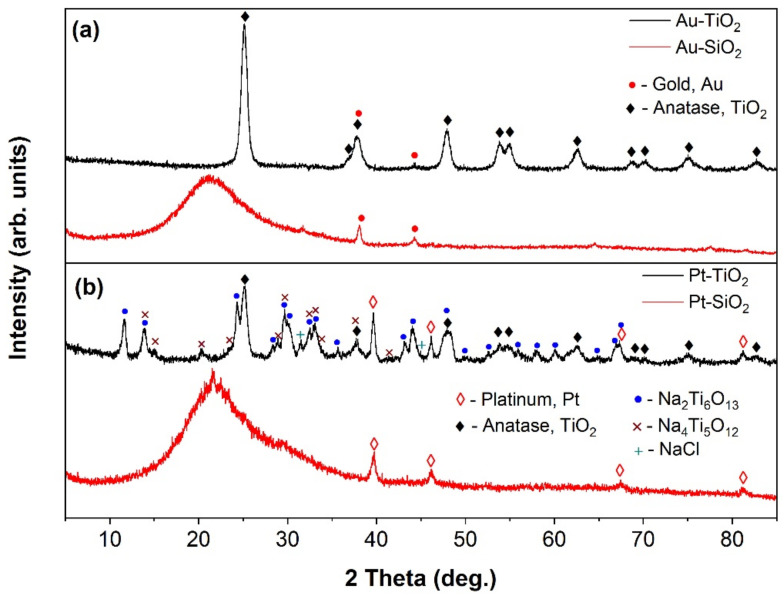
XRD patterns of the samples; (**a**) Au- and (**b**) Pt-doped samples.

XRD and XRF analyses were performed to confirm the presence of the dopants in the TiO_2_ and SiO_2_ matrices, revealing the existence of Au and Pt crystalline phases in the samples. Figure 4a,b display the diffractograms of Au-doped and Pt-doped samples, respectively. Two crystalline phases were observed in the Au-doped TiO_2_ (AuTiO_2_) sample: metallic Au and anatase TiO_2_. The Au and anatase phases were identified based on the ICDD file no. 4-0784 and no. 21-1272, respectively. The RIR method was employed to calculate the quantity of Au relative to anatase TiO_2_ and was estimated to be 1.92%. 

The Au-doped SiO_2_ sample (AuSiO_2_) contains a metallic Au phase and an amorphous SiO_2_ matrix. The PtSiO_2_ sample, on the other hand, turned out to be similar to the AuSiO_2_ sample, with an amorphous SiO_2_ matrix and a metallic Pt phase detected by XRD using ICDD file no. 4-0802. The amount of platinum identified by XRF analysis was 0.46% by mass. The Pt-TiO_2_ sample was noted to have a complex composition, with the identification of anatase TiO_2_, Pt, and two sodium titanates (Na_2_Ti_6_O_13_ and Na_4_Ti_5_O_12_) based on ICDD files nos. 14-0277 and 37-0273, respectively. Additionally, a small amount of NaCl was detected. The XRF analysis of the PtTiO_2_ sample indicated a platinum quantity of 0.74% by mass. According to the XRD analysis, the crystallite sizes of metals are: 10 nm AuTiO_2_, 27 nm AuSiO_2_, 18 nm PtTiO_2_, and 14 nm PtSiO_2_.

### 2.4. X-ray Photoelectron Spectroscopy

**Figure 5:**
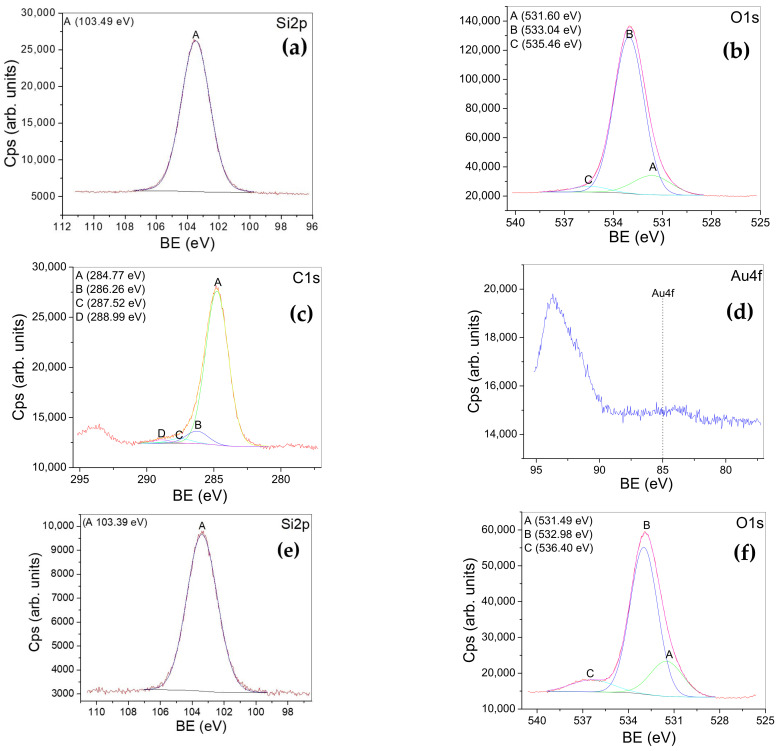

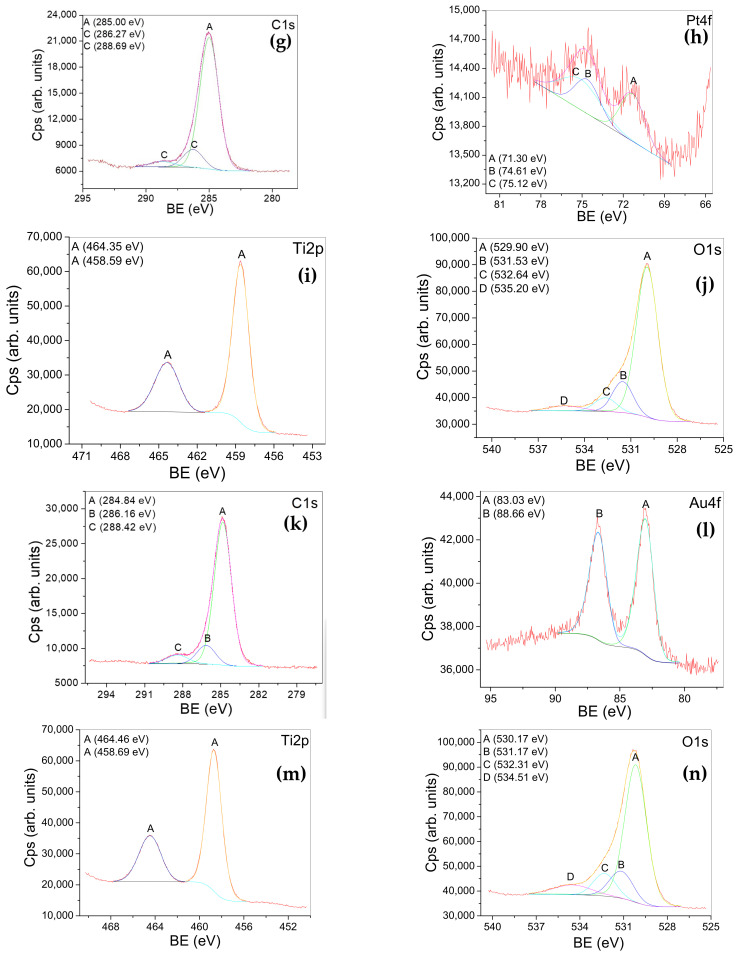

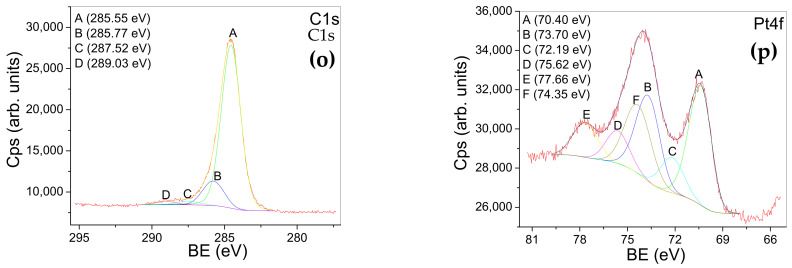
XPS high-resolution spectra (Si2p, O1s, C1s, Au4f, and Pt4f lines) registered for the samples: Au-SiO_2_ (**a**–**d**), Pt-SiO_2_ (**e**–**h**), Au-TiO_2_ (**i**–**l**), and Pt-TiO_2_ (**m**–**p**).

Figure 5 illustrates the high-resolution spectra for the elements (Si2p, Ti2p, Au4f, Pt4f, and C1s) in the samples: AuSiO_2_ (Figure 5a–d), PtSiO_2_ (Figure 5e–h), AuTiO_2_ (Figure 5i–l), and PtTiO_2_ (Figure 5m–p).

The registered XPS lines are assigned according to Table 1.

### 2.5. UV–Vis Characterization

In order to reveal the light absorptive properties of the investigated materials, the UV–Vis characterization was performed on SiO_2_ and TiO_2_ samples before and after their modification as well as for PVA-based gels containing the mentioned powders. Accordingly, the UV–Vis spectra were recorded for the following samples (Table 2).

**Figure 6:**
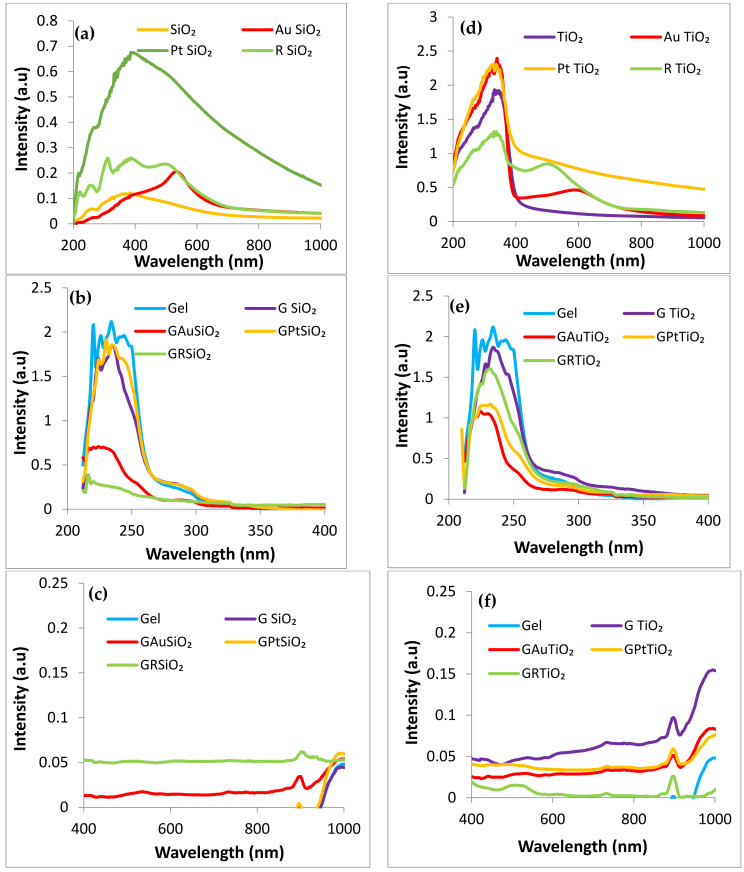
Comparative UV–Vis spectra of bare and modified SiO_2_ and TiO_2_ powders also embedded in PVA gel: SiO_2_-based powders in the range of 200–1000 nm (**a**), gels containing powders based on SiO_2_ in the range of 200–400 nm (**b**), gels containing powders based on SiO_2_ in the range of 400–1000 nm (**c**), TiO_2_-based powders in the range of 200–1000 nm (**d**), gels containing powders based on TiO_2_ in the range of 200–400 nm (**e**), gels containing powders based on TiO_2_ in the range of 400–1000 nm (**f**).

The spectra were recorded between 200 and 1000 nm (Figure 6a,d) and represent the gel-containing samples in the 200–400 nm (Figure 6b,e) and 400–1000 nm (Figure 6c,f) for a better evaluation of their light absorptive properties in the UV and Vis domains.

Tubular SiO_2_ (SiO_2_ sample) shows a broad absorption band spanning from the UV to the visible domain (300–600 nm, Figure 6a), which is amplified by modifiers and red-shifted. The presence of gold (AuSiO_2_ sample) induces the appearance of the surface plasmon resonance phenomenon (peak centered at 550 nm), while the addition of ruthenizer (RSiO_2_ sample) results in a maximum absorption peak around 520 nm. Furthermore, platinum nanoparticles (PtSiO_2_ sample) significantly enhance the light absorption capacity of the sample. The TiO_2_ sample exhibits an absorption edge at around 400 nm. These considerations also apply to the TiO_2_-based samples, where the presence of gold (AuTiO_2_ sample) and ruthenizer (RTiO_2_ sample) is marked by well-defined peaks in the visible domain. In the case of gold, there is a slight red shift relative to SiO_2_.

Except for the silica modified with ruthenizer and embedded in gel (GRSiO_2_ sample), the light absorption in the UV domain of the gel-containing powders occurs between 200 and 250 nm and is lower (Figure 6b,e) than for the bare gel (Gel sample) but higher than for the oxide powders in their standalone form. Although the PVA-DMSO (dimethyl sulfoxide) gel proved significant transparency in the range of 400–700 nm [11], the powders embedded in gels show no light absorption in the visible domain (Figure 6c,f). 

### 2.6. ROS Photogeneration

i. Generation of hydroxyl radicals (•OH) under light irradiation

The presence of hydroxyl radicals (•OH) was checked in a coumarin aqueous solution containing the samples of interest (powders or gels) and exposed to simulated solar light. The same tests have been performed for visible light irradiation, but all the investigated samples proved to be without activity. In the case of solar light utilization, some active samples generate (•OH) that leads after reaction with coumarin to the umbelliferone formation, a fluorescent product signaled by a PL emission at 451 nm for λexc = 330 nm [34].

**Figure 7:**
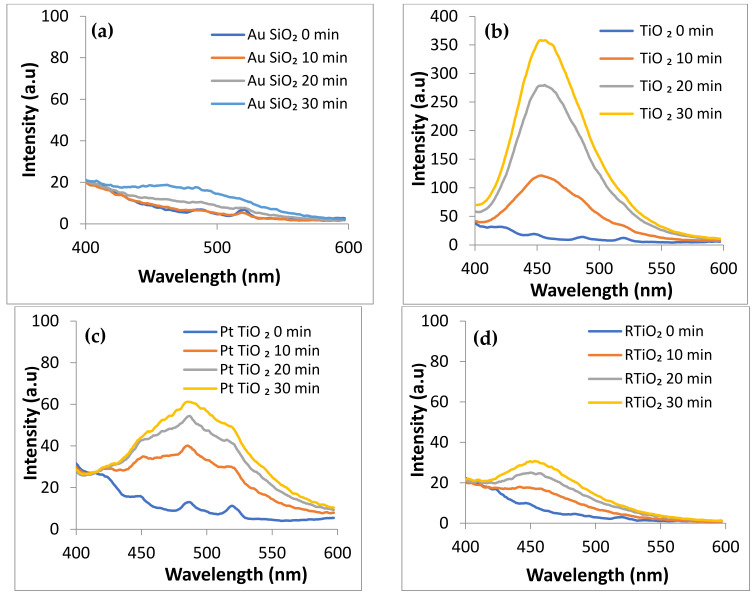

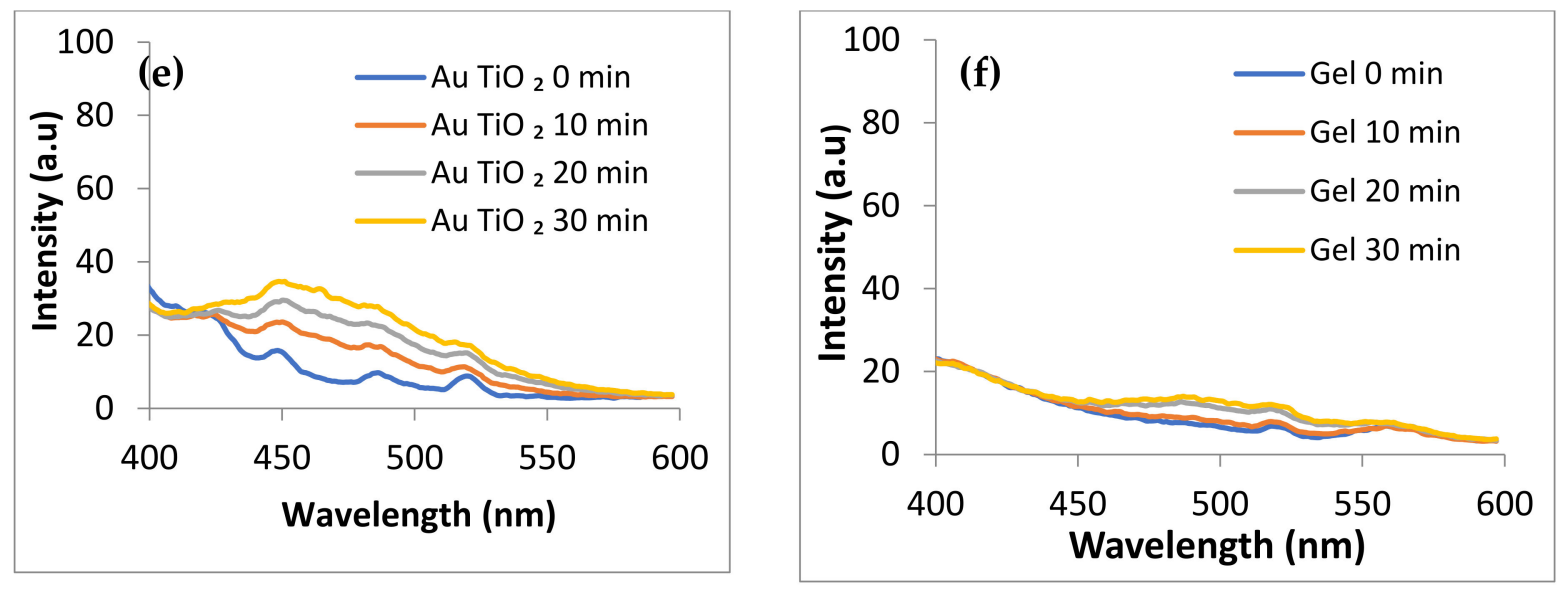
The generation of the hydroxyl radicals under simulated solar irradiation in the presence of: AuSiO_2_ powder (**a**), AuTiO_2_ powder (**b**), PtTiO_2_ powder (**c**), RTiO_2_ powder (**d**), AuTiO_2_ powder (**e**), PVA gel (**f**).

No significant activity for the SiO_2_-based samples was registered, with only small amounts of hydroxyl radicals being produced after 30 min of irradiation by the AuSiO_2_ powder sample (Figure 7a). Also, the GSiO_2_ sample (SiO_2_ embedded in PVA gel) proved a slight tendency in this sense, probably due to the gel properties (Supplementary Information—Figure S4). TiO_2_-based samples show a higher capacity to generate hydroxyl radicals under solar irradiation, according to the sequence TiO_2_ > PtTiO_2_ >RTiO_2_ > AuTiO_2_ (Figure 7b–e) but this disappears after mixing with gel (Supplementary Information—Figure S4). The PVA gel produces only traces of •OH (Figure 7f).

Hydroxyl radical is known as a powerful oxidant agent, acting on various organic biomolecules [12,36]. Accordingly, the identified samples as •OH providers under solar light exposure could act as antibacterial agents too.

ii. Generation of Singlet Oxygen (^1^O_2_) under visible irradiation (λ > 420 nm) monitored by using Singlet Oxygen Sensor (SOSG) λexc = 480 nm

**Figure 8:**
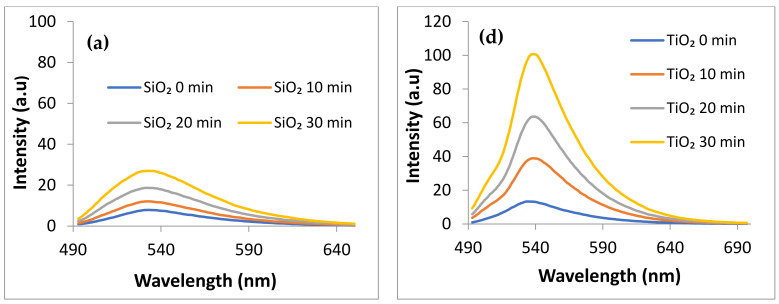

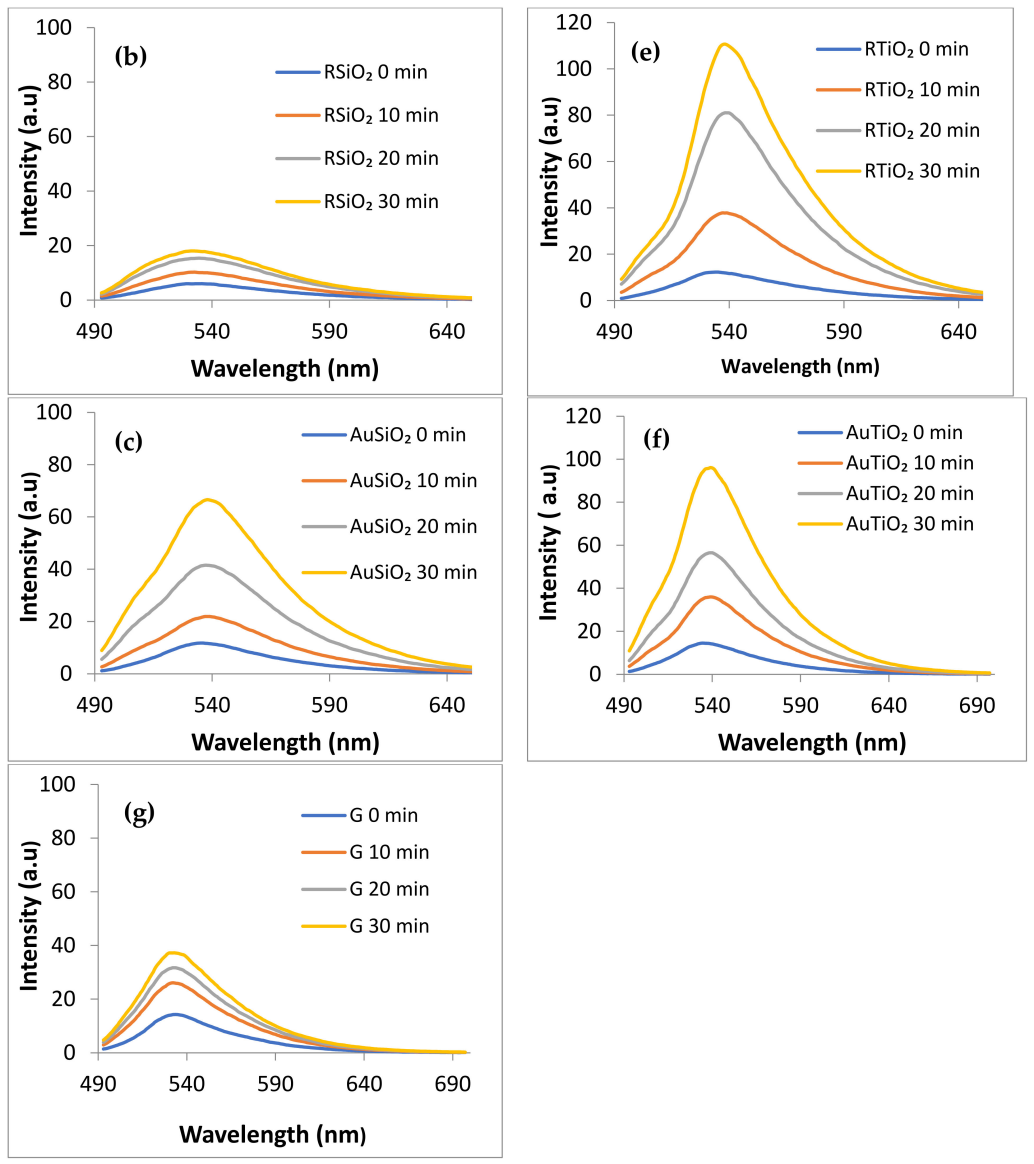
Monitoring of singlet oxygen generation under visible light irradiation by using Singlet Oxygen Sensor (SOSG), λexc = 480 nm, in the presence of: SiO_2_ powder (**a**), RSiO_2_ powder (**b**), AuSiO_2_ powder (**c**), TiO_2_ powder (**d**), RTiO_2_ powder (**e)**, AuTiO_2_ powder (**f**), PVA gel (**g**).

The generation of oxygen singlet particularly for applications in the biomedical field, especially such as photodynamic therapy, has received significant interest. The target samples, when exposed to a methanolic solution of SOSG, produce photogenerated oxygen singlet (^1^O_2_), leading to the formation of endoperoxide. This process is indicated by a peak in photoluminescence (PL) emission around 530 nm. The time course of ^1^O_2_ generation by SiO_2_ powder is illustrated in Figure 8a, clearly showing an increasing amount over time. While the addition of ruthenizer does not contribute to singlet oxygen generation (Figure 8b), the incorporation of gold greatly enhances this process (Figure 8c).

The oxygen singlet generation by the highly defected SiO_2_ was previously reported by our group [35] but the discovery of its improvement through the addition of gold is a novel finding. Unlike this, modification with gold of the TiO_2_ does not significantly change its activity (Figure 8f) but a small increase in ^1^O_2_ generation is obtained for the ruthenizer-modified TiO_2_ (RTiO_2_ sample) (Figure 8e). Our previous work also reported the TiO_2_ ability to generate oxygen singlet under visible irradiation without improvements brought by Au and Ag nanoparticles [37].

Since ^1^O_2_ induces bio-membrane degradation [12], a correlation of these data with the antibacterial activity is of great importance for practical applications. The PVA (gel sample) shows a significant activity for ^1^O_2_ generation (Figure 8g).

### 2.7. Electrokinetic Potential Measurements

Electrokinetic potential measurements are meant to investigate the charged surface of the engineered nanoparticles in water and the presumable interaction with bacterial cell surface that usually bears a negative net charge at pH 7. Table 3 shows the negative zeta potential for both oxide powders (SiO_2_ and TiO_2_) that shift positively by mixing with gel whose zeta potential is quite close to 0. The experiments were made in triplicates.

The modification of SiO_2_ with metals results in a decrease in zeta potential values, with platinum-modified SiO_2_ (PtSiO_2_) reaching as low as −60 mV. In contrast, the addition of metals to the TiO_2_ samples increases the potential values. Based on these findings, it can be inferred that the surface charge of the gel and gel containing TiO_2_ powders is closest to establishing contact with bacterial cells.

### 2.8. Antibacterial Activity Assays of the Investigated Samples against S. aureus

#### 2.8.1. Antibacterial Activity Assays in Dark

**Figure 9:**
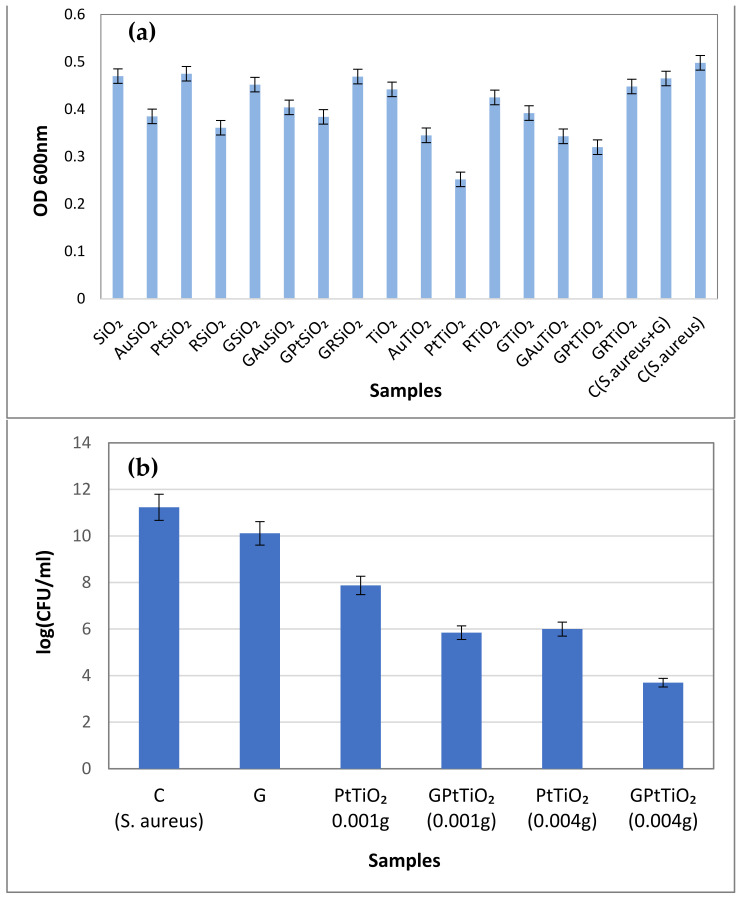
Antibacterial activity of all investigated samples (0.001 g) against *S. aureus* in the dark, quantified as the optical density (**a**); cell viability (logarithm of the colony forming units) over gel sample and different amounts of PtTiO_2_ materials, in standalone forms or embedded in gel (**b**). The experiments were made in triplicates.

The antibacterial activity assay of the investigated samples against *S. aureus* is presented in Figure 9. Figure 9a allows comparing the antimicrobial activity of the investigated samples in the dark, leading to a hierarchization of the target materials. Both unmodified SiO_2_ and TiO_2_ samples show insignificant antibacterial activity (Figure 9a) which is consistent with the reported data [24]. The lowest cellular viability relative to the control (*S. aureus*) was registered for the PtTiO_2_ sample, the gel embedding of PtTiO_2_ powder has a lower antibacterial effect. Therefore, the notable reduction in microbial growth induced by the Pt-TiO_2_ sample (Figure 9a) can be assigned to the presence of platinum nanoparticles in the TiO_2_ matrix and to their interaction. The antibacterial activity of platinum nanoparticles against Gram-negative and Gram-positive bacteria (such as *E. coli* and *S. aureus*) is well-documented [15,16,18,38]. Ahmed et al. [17] reported the use of PtNPs 2–5 nm in size for reducing bacterial cell viability through reactive oxygen species (ROS) production and membrane integrity loss. But, according to Aygun et al. [18], ROS formation can also be induced in mammalian cells by the platinum nanoparticles, and their use for therapeutic purposes requires more data. 

Supplementary data are provided in Figure 9b concerning the antibacterial effect of PtTiO_2_-based materials in the dark. A sharp decrease in logarithm in colony-forming units is depicted for PtTiO_2_ powder when increased the inorganic material amount (from 0.001–0.004 g). Also, the gel sample proves intrinsic antibacterial activity, therefore enhancing the antibacterial effect of the embedded PtTiO_2_ powder. Table 3 indicates a negative electrokinetic potential for the PtTiO_2_ material, suggesting that bacterial cell adhesion to the PtTiO_2_ sample could be hindered. Thus, it is reasonable to assume that other antibacterial mechanisms, such as reactive oxygen species (ROS) generation and ion release, are at play. Unlike this, the antibacterial effect of PtSiO_2_ material is almost insignificant, the morphological and structural properties of the deposited metal nanoparticles proving to be dependent on the support characteristics. Although it has lower antibacterial reactivity, AuTiO_2_ seems to be closer to PtTiO_2._ The unsupported Au nanoparticles also emphasize antibacterial activity in the dark, the accumulation of AuNPs on the cell wall, their diffusion, and the resulting cell lysis is more pronounced in Gram-negative (*E. Coli*) than in Gram-positive bacteria (*S. aureus*) [15,39].

#### 2.8.2. Antibacterial Activity Assays under Light Exposure

**Figure 10:**
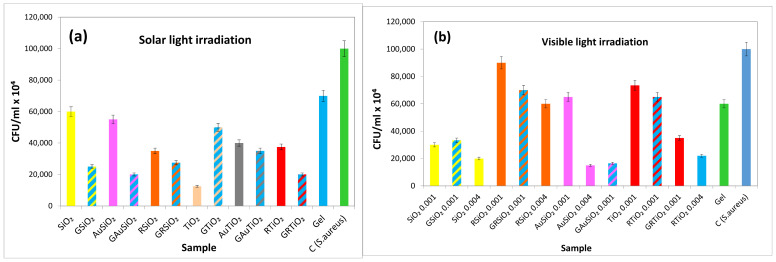
Antibacterial activity of the selected samples (0.001 g) against *S. aureus* under solar light exposure (AM 1.5) for 30 min (**a**); antibacterial activity of the selected samples (0.001 g and 0.004 g) under visible light exposure (λ > 420 nm) for 30 min (**b**). The experiments were made in triplicates.

According to Figure 10a, bacterial cell viability is primarily reduced in the presence of a TiO_2_ sample exposed to solar light irradiation. This aligns with the findings in Figure 7b which confirms the ability of TiO_2_ to photogenerate hydroxyl radicals. The formation of singlet oxygen is also conceivable, considering that the cell culture medium contains both D_2_O and H_2_O. The TiO_2_-based powders exhibit superior activity compared to the SiO_2_ ones. While adding gel to bare TiO_2_ does not provide any benefits, it leads to a slight decrease in cell viability for its derivative samples (GAuTiO_2_, GRTiO_2_). The gels containing SiO_2_-based materials show better activity than free powders under solar light exposure.

Figure 10b particularly highlights the antibacterial activity of SiO_2_ and AuSiO_2_ samples against *S. aureus* under visible light irradiation. Since SiO_2_ does not exhibit any antibacterial activity in dark conditions, this behavior can be attributed to the photo generation of the reactive oxygen species. Figure 8a,c confirms that SiO_2_ but especially the AuSiO_2_ sample produce oxygen singlet when exposed to visible light. The enhanced singlet oxygen production was achieved using D_2_O instead of H_2_O, which generally acts as a quencher for singlet oxygen. By incorporating gold nanoparticles as modifiers in the SiO_2_ matrix, an improvement in light absorption and activation of surface defects was achieved, leading to increased singlet oxygen production as the primary antibacterial mechanism. The ruthenizer used as a sensitizer in the case of TiO_2_ samples proves to be efficient too.

Comparing Figure 10a,b, a better antibacterial activity of the bare SiO_2_ appears under visible compared to solar light irradiation. This may initially seem contradictory since, by exposure to solar irradiation, SiO_2_ can utilize the light energy in the UV range (corresponding to the solar spectrum), not only from the visible spectrum. However, this result can be explained by the higher production of singlet oxygen, favored during the tests containing only D_2_O (without water). Unlike this, under solar light exposure, hydroxyl radicals are predominantly generated, which explains the higher activity of the TiO_2_ sample (Figure 10a). The antibacterial assays conducted under visible light exposure and depicted in Figure 10b, indicate the addition of gold to silica as the best modification for oxide matrices. Despite the enhanced light absorption, the addition of ruthenizer does not significantly reduce microbial growth observed for SiO_2_. In the case of TiO_2_, the decrease in cell viability with the increase in the modified sample (0.001–0.004 g RTiO_2_) can be due to the presence of a ruthenizer.

Therefore, several additional considerations can be made regarding the AuSiO_2_ sample when exposed to light. UV–Vis measurements reveal that the bare oxides (SiO_2_ and TiO_2_) exhibit light absorption properties that cover the UV–Vis and UV domains. The modification of these matrices with metals increases light absorption and induces surface plasmon resonance, consistent with the reported literature [40,41]. The presence of gold is observed in both SiO_2_ and TiO_2_ but its effect on enhancing oxygen singlet generation is only evident in the case of Au-SiO_2_ sample. This observation aligns well with the antibacterial activity exhibited by the AuSiO_2_ sample against *S. aureus* under visible light irradiation. In this case, the antibacterial assay was conducted in the presence of D_2_O, without H_2_O, to specifically highlight the formation of singlet oxygen. Consequently, the better antibacterial effect observed for the AuSiO_2_ sample can be attributed to the presence of singlet oxygen which is promoted by the presence of gold in the SiO_2_ matrix. The bioactivity of gold nanoparticles is well-established and extensively studied [42,43].

This result is significant as it demonstrates the improvement in oxygen singlet photogeneration compared to the bare sample and it is achieved under visible light irradiation, unlike similar studies that employ UV irradiation for both SiO_2_ and TiO_2_ [12]. 

Although many silica-based materials have been tested for their antimicrobial properties [44,45], various modifiers have been used since the bare SiO_2_ did not indicate intrinsic antimicrobial properties, neither in dark [46,47,48] nor under light exposure [49]. Based on these considerations, it can be assumed an advance in the biomaterials field triggered by this result: the endowing of an inactive material, namely SiO_2_, with antibacterial properties by exposing it to visible light irradiation. More than that, the antibacterial capacity can be increased by modifying the SiO_2_ matrix with gold nanoparticles due to the singlet oxygen generation. This aspect is all the more important as it is known the ability of metal nanoparticles to induce oxidative stress on the bacterial cell, the singlet oxygen is responsible to a greater extent for cell damage than the super anion radical and hydrogen peroxide (endogenous antioxidants can easier reduce their effect) [19].

## 3. Conclusions

The present work highlights the development of an efficient antibacterial agent against *S. aureus* in the dark, specifically PtTiO_2_ powder. This goal is achieved by modifying previously synthesized sol–gel TiO_2_ nanoparticles with glutathione-capped platinum nanoparticles. Additionally, the obtained PVA hydrogel exhibits intrinsic antibacterial activity and acts as an effective dispersive medium for the PtTiO_2_ powder, enhancing its antibacterial behavior.

The results of antibacterial assays conducted under visible light irradiation reveal the potential of highly defected SiO_2_ with tubular morphology as an optically active material capable of reducing the cell viability of *S. aureus* through the photogeneration of singlet oxygen. Moreover, the antibacterial properties of SiO_2_ can be further improved by modifying its matrix with glutathione-capped gold nanoparticles.

These findings represent significant progress towards SiO_2_ and AuSiO_2_ applications in the biomedical field (e.g., photodynamic therapy) owing to their ability to generate singlet oxygen under visible light irradiation.

## 4. Materials and Methods

### 4.1. Synthesis of Materials

#### 4.1.1. Synthesis of SiO_2_ and TiO_2_ Matrices

SiO_2_ with tubular morphology was synthesized by using a modified sol–gel method, according to previously reported works [33,34,35]. DL tartaric acid (TA, Riedel de Häen, Buchs SG, Switzerland) was dissolved in ethanol absolute (99.5% Merck) and ultrapure water. For the template formation, namely ammonium tartrate, gaseous ammonia was bubbled in the above-mentioned mixture. TEOS (tetraorthosilicate, 99%, AlfaAesar) was slowly added according to the molar ratio: 1 TEOS/0.035 TA/21.5 C_2_H_5_OH/18 H_2_O. The resulting gel was filtered, dried, and thermally treated in air at 500 °C for 3 h.

TiO_2_ nanoparticles were obtained by sol–gel method using titanium isopropoxide (97%, Aldrich, Steinheim, Germany) and 2, 4-Pentanedione (Alfa Aesar, Karlsruhe, Germany) according to the reported paper [50]. The gel aging was allowed before drying and thermal treatment at 400 °C, in air, for 1 h. Subsequent hydrothermal treatment was applied in order to increase the defect concentration, as follows: the obtained powder was introduced in an autoclave containing hydrazine monohydrate (N_2_H_4_×H_2_O 98%, Alfa Aesar) and aqueous solution of NaOH (Alfa Aesar) for 1 h at 140 °C.

#### 4.1.2. Glutathione Capped Gold Nanoparticles 

They were obtained by adapting the synthesis route reported by Wu et al. [51]. Gold (III) chloride trihydrate (HAuCl_4_×3H_2_O ACS reagent > 49% Au) was dissolved in ultrapure water Millie-Q system, >18 MΩcm (24 mM) and kept at 0 °C under stirring. 

L-Glutathione reduced (GSH, > 98%, Carl Roth, 0.2 mmol) was dissolved in 100 mL ultrapure water Millie-Q system, >18 MΩcm, and slowly added to the gold solution. The clear mixture was kept under gentle stirring for 1 h. Sodium borohydride solution (NaBH_4_ Sigma-Aldrich, 12 wt. % in 14 M NaOH) was added to the above-mentioned mixture that changes into a dark red solution that is added after 24 h to the annealed SiO_2_ and TiO_2_ powders. These are dried (30 min, 100 °C) and then thermally treated in the air for 3 h at 600 °C.

#### 4.1.3. Glutathione Capped Platinum Nanoparticles 

They were obtained by adapting the synthesis route reported by Eklund [52]. Chloroplatinic acid hexahydrate (H_2_PtCl_6_×6H_2_O, Sigma-Aldrich) was dissolved in ultrapure water (12 mM) and subjected to a similar procedure to the previous one. Unlike that, the aqueous mixture GSH—H_2_PtCl_6_ turns in yellow color when added NaBH_4_ solution.

#### 4.1.4. Modification of SiO_2_ and TiO_2_ with Ruthenium Dye

Ruthenizer (535-bis TBA from Solaronix, Aubonne, Switzerland) was used to obtain the sensitized oxides (RSiO_2_ and RTiO_2_) for increasing the generation of singlet oxygen under light irradiation. In this sense, Ruthenizer (0.0005 g in 0.5 mL ultrapure water) was added to 0.015 g annealed oxide powders (SiO_2_, TiO_2_) and then dried at 60 °C.

#### 4.1.5. PVA Hydrogel Synthesis

It follows the route reported by Hou et al. [11]. Briefly, 0.04 g Poly (vinyl alcohol) powder (Sigma-Aldrich, 80% hydrolyzed) was dissolved in 1 mL dimethyl sulfoxide (DMSO, Life Technologies, MW 78.13) and 0.5 mL H_2_O/D_2_O). In the case of gel samples exposed to visible light irradiation (for oxygen singlet checking), H_2_O is replaced by D_2_O. For UV–Vis characterization, 0.02 g oxide powder (bare and modified SiO_2_ and TiO_2_) is added to the freshly prepared gel aliquots (2 mL), and for antibacterial tests 0.001 g oxide powder is mixed with 10 μL gel sample. The resulting mixtures are gently shaken for 30 min.

### 4.2. Characterization of Materials

#### 4.2.1. Electron Microscopy Characterization

SEM images have been recorded with an FEI Quanta 3D microscope, FEG model. Morpho-structural characterization was carried out using JEM ARM200F Analytical Transmission Electron Microscope (JEOL Ltd., Tokyo, Japan).

#### 4.2.2. AFM Characterization

Atomic force microscopy (AFM) measurements were performed with a microscope produced by Park Systems (XE-100 model) working in non-contact mode. The microscope allows obtaining accurate images due to its configuration, namely decoupled XY/Z, flexure-guided cross talk eliminated scanners. In all AFM experiments sharp tips with less than 10 nm radius of curvature, ~125 μm length, ~30 μm width, ~42N/m force constant, and ~330 kHz resonance frequency (NCHR, Nanosensors™) were used. The AFM images were processed with the XEI program (v 1.8.0—Park Systems, Suwon, Korea) for thermal tilt correction and roughness assessment. The root mean squared roughness (Rq) represents the standard deviation of the height value in the image and the peak-to-valley parameter (Rpv) means the height difference between minimum and maximum. The AFM images are presented in classic mode (monochrome z-scale gradient) accompanied by representative line scans (plot of random line scans, each AFM image consisting of 256 lines) which show the surface profile in detail. 

#### 4.2.3. XRD and XRF Measurements

The Rigaku Ultima IV multipurpose diffraction system (Rigaku Corp., Tokyo, Japan) was used to obtain XRD patterns, employing a Cu-target tube (λ = 1.54060 Å). The working conditions included 30 mA and 40 kV, and the data were collected at room temperature within a 2θ range of 5 to 85°, with a step size of 0.02° and a scan rate of 2°/min. For the elemental analysis of the materials (XRF measurement), the Rigaku ZSX Primus II spectrometer (Rigaku Corp., Tokyo, Japan) with wavelength dispersion in a vacuum was utilized. Rigaku’s SQX analytical software was employed for semi-quantitative standardless analysis to compute the chemical composition of the samples.

#### 4.2.4. XPS Characterization

XPS investigations were performed using a SPECS spectrometer with a PHOIBOS (150) analyzer equipped with non-monochromatized Mg Kα (1253.6 eV) X-rays anode radiation source operated at 300 W. Wide and detail spectra were registered at pressures lower than 2 × 10^−9^ mbar, pass energy of 50 eV and 20 eV. The C1s peak was set at 285.00 eV for the binding energy scale. The spectra were fitted using Voigt peak profiles and a linear or a Shirley background (depending on shape), using the SDP v7.0 software (XPS International, Salem, Oregon, United States).

#### 4.2.5. Diffuse Reflectance UV–Vis Characterization 

Diffuse reflectance UV–Vis spectra were obtained using a spectrophotometer Perkin Elmer Lambda 35, equipped with an integrating sphere. The measurements were carried out in the range of 1100–250 nm, using Spectralon as a reference. The reflectance measurements were converted to absorption spectra using the Kubelka–Munk function, F(R). For determining the UV–Vis light absorption properties of the gels, the scattered transmission was measured using the same instrument (Perkin Elmer, Lambda 35). The sample was placed in micro-cuvettes (Brand: 12.5 mm × 12.5 mm × 45 mm, center height: 8.5 mm, 230–900 nm, Roth) and introduced in the transmission port of the integrating sphere. The transmitted and forward scattered light was collected and measured by the sphere (during the measurements the Spectralon was placed in the reflectance port of the instrument).

#### 4.2.6. Monitoring of ROS Photogeneration

(i) Singlet oxygen (^1^O_2_) formation under visible light exposure

Target samples (0.001 g powder or 0.5 mL gel) were suspended in methanolic solution of SOSG (Singlet Oxygen Sensor Green–Thermo Fisher Scientific/Invitrogen) contained by quartz cuvettes and exposed to visible irradiation (provided by a Peccel solar simulator equipped with cut off filter λ > 420 nm Asahi Spectra). The photogenerated oxygen singlet reacts with a SOSG component leading to the endoperoxide formation signaled by the appearance of the photoluminescence peak around 530 nm [35,53,54] (λexc = 480 nm, excitation and emission slits 2.5/2.5). This is depicted with a Carry Eclipse fluorescence spectrometer, Agilent Technologies.

(ii) Hydroxyl radicals (•OH) generation under simulated solar light

The samples of interest (0.001 g powder or 0.5 mL gel) were suspended in a coumarin solution (10 mM, Merck) and exposed to simulated solar irradiation (Peccel Solar Simulator, Tokyo, Japan). The photogenerated hydroxyl radicals react with coumarin leading to the formation of a fluorescent product, umbelliferone, that is evaluated according to a characteristic PL emission peak from 450 nm for λexc = 330 nm [55,56] (excitation and emission slits 5/10, Carry Eclipse fluorescence spectrometer). 

#### 4.2.7. Electrokinetic Potential Measurements

Electrokinetic potential measurements have been performed on a Malvern Nano ZS Zetasizer, Model ZEN 3600 at room temperature. The measurements were performed in triplicate, the values being averaged.

#### 4.2.8. Antibacterial Activity

The antimicrobial activity of the investigated samples (powders and gels containing powders) was assessed against the bacterial strain Staphylococcus (*S.aureus* ATCC 29213). The bacterial strain was grown in LB liquid medium (g/L: peptone 10 g, NaCl 5 g, yeast extract 5 g, pH 6) together with the samples of interest (under light exposure or in dark, at 37 °C). 

Firstly, the cell growth in the dark was evaluated by measuring the optical density (OD) at 600 nm with the multireader Bio Tek Synergy HTX, Agilent US. All tests were performed in triplicate.

To highlight the dependence of the bacterial cells’ viability after 24 h on the sample characteristics and testing conditions (type of irradiation, powder amount), the decimal dilutions assay was performed. The successive decimal dilutions (1:10 *v*/*v*) are necessary to obtain a less dense suspension that is scattered on solid nutritional broth. After incubation on optimal conditions (37 °C, 18–24 h) the number of isolated colonies is counted, and the UFC/mL value is determined by multiplying with the dilution factor (Ben-David A, 2014). *S. aureus* ATCC 29213 grown in LB liquid broth was used as control. For samples exposed to visible light irradiation, the bacterial cell culture contained D_2_O instead of H_2_O and for solar irradiation contained D_2_O and H_2_O.

## Figures and Tables

**Table 1:** The assignments of the XPS lines.

Sample	Si2p/Ti2p	O1s	C1s	Au4f/Pt4f
AuSiO_2_	Peak A 103 eV-assigned to Si^4+^state (SiO_2_) (Figure 5a)	Peak A-531.60 eV -assigned to: bonds with carbon, adsorbed oxygen, and sodium carbonates Peak B-533.04 eV -assigned to oxygen in SiO_2_ Peak C-535.46 eV -assigned to Na Auger (Figure 5b)	Peak A-284.77 eV -assigned to: C-C and C-H bonds Peak B-286.26 eV -assigned to C-O bonds PeakC-287.52 eV -assigned to: C=O and O-C-O bonds Peak D 288.99 eV -assigned to: O-C=O (COOH, COOR) bonds (Figure 5c)	Au4f -assigned to a small amount of Au on the surface of the sample (Figure 5d)
PtSiO_2_	Peak A 103 eV -assigned to Si^4+^state (SiO_2_) (Figure 5e)	Peak A 531.49 eV -assigned to: metal hydroxide (NaOH), O=C bonds, molecularly adsorbed oxygen PeakB 532.98 eV -assigned to oxygen in SiO_2_ bonds with carbon Peak C 536.40 eV -assigned to Na Auger (Figure 5f)	Peak A 285.00 eV -assigned to: C-C and C-H bonds PeakC- 286.27 eV PeakC- 288.69 eV -assigned to: C-O and O-C=O bonds (Figure 5g)	A-B doublet (71.3;74.61eV) -assigned to metallic Pt PeakC- 75.12 eV -assigned to the loss from Na 2s (Figure 5h)
AuTiO_2_	Peak A 464.35 eV Peak A 458.59 eV (2p3/2;2p1/2) -assigned to Ti in TiO_2_ (Figure 5i)	Peak A-529.90 eV -assigned to oxygen chemical states bonded in TiO_2_ Peak B-531.53 eV -assigned to: O=C _(adsorbed)_ bonds, the presence of metal hydroxide (NaOH) Peak C-532.64 eV -assigned to O-C bonds Peak D-535.20 eV -assigned to Na Auger transition (Figure 5j)	Peak A 284.84 eV -assigned to: C-C and C-H bonds Peak B 286.16 eV -assigned to C-O bonds Peak C 288.42 eV -assigned to O-C=O (COOH and COOR) bonds (Figure 5k)	Au4f Peak A 83.03 eV Peak B 88.66 eV (4f7/2; 4f5/2) -the binding energy is lower than that of ordinary metal, suggesting that Au receives electrons. (Figure 5l)
PtTiO_2_	Peak A 464.46 eV Peak A 458.69 eV (2p3/2;2p1/2) -assigned to Ti in TiO_2_ (Figure 5m)	Peak A 530.17 eV -assigned to oxygen chemical states bonded in TiO_2_ Peak B 531.17 eV -assigned to: O=C bonds; metal hydroxide (NaOH) Peak C 532.31 eV -assigned to O-C bonds Peak D 534.51 eV -assigned to Na Auger transition (Figure 5n)	Peak A 285 eV -assigned to: C-C and C-H bonds Peak B 285.77 eV -assigned to C-O bonds Peak C 287.52 eV Peak D 289.03 eV -assigned to: O-C-O, O-C=O (COOH and COOR) bonds (Figure 5o)	A-B doublet (70.4; 73.7 eV) -assigned to metallic Pt (suggesting an induced negativity) C-D doublet (72.1, 75.6 eV) -assigned to Pt(OH)_2_ or a Pt sub-oxide F-E doublet (74.3; 77.6 eV) -assigned to PtO and PtO_2_. (Figure 5p)

**Table 2:** Nomenclature of the samples.

Powder	SiO_2_	AuSiO_2_	PtSiO_2_	* RSiO_2_	TiO_2_	AuTiO_2_	PtTiO_2_	* RTiO_2_
PVA gel containing oxide powders	GSiO_2_	GAuSiO_2_	GPtSiO_2_	GRSiO_2_	GTiO_2_	GAuTiO_2_	GPtTiO_2_	GRTiO_2_
PVA gel	Gel (G)							

* RSiO_2_ and RTiO_2_ denote the powders modified with ruthenizer.

**Table 3:** Electrokinetic potential values for SiO_2_ and TiO_2_-based powders in standalone form and embedded in PVA gel.

Powder Samples	Electrokinetic Potential (mV)	Gel Embedding Powder Samples	Electrokinetic Potential (mV)
		Gel	−1.10
SiO_2_	−14.60	GSiO_2_	−6.00
AuSiO_2_	−17.73	GAuSiO_2_	−4.81
PtSiO_2_	−60.43	GPtSiO_2_	−18.00
RSiO_2_	−17.40	GRSiO_2_	−7.58
TiO_2_	−30.00	GRTiO_2_	–0.99
AuTiO_2_	−17.63	GAuTiO_2_	−2.07
PtTiO_2_	−25.60	GPtTiO_2_	−4.61
RTiO_2_	−15.33	GRTiO_2_	−0.99

## Data Availability

Data were included in the manuscript. Supplementary information can be obtained by request to the corresponding authors.

## Supplementary Materials

The following supporting information can be downloaded at: https://www.mdpi.com/article/10.3390/gels9080650/s1, Figure S1: Topographic 2D AFM image of the TiO_2_ powder modified with AuNPs; Figure S2: Part (slice) of a SiO_2_ tube exhibited in a 3D topographic AFM image o at the scale of 4 μm × 25 μm; Figure S3: Part of a SiO_2_ tube exhibited in a 3D topographic AFM image o at the scale of 6 μm × 8 μm; Figure S4: The generation of the hydroxyl radicals under simulated solar irradiation by SiO_2_ and TiO_2_ based powders, free and embedded in PVA gel; Figure S5: Electrokinetic potential measurements for SiO_2_ and TiO_2_-based powders free and embedded in PVA gel. The experiments were made in triplicates.
