# Supplementary material for: Antibacterial Activity of PVA Hydrogels Embedding Oxide Nanostructures Sensitized by Noble Metals and Ruthenium Dye
Source: Gels. 2023 Aug 11;9(8):650. doi: 10.3390/gels9080650 (PMC10454060; doi:10.3390/gels9080650)
Supplement: Supplementary file 1 [file gels-09-00650-s001.zip › gels-2427032-supplementary.pdf]

gels

MDPI

Supplementary Materials

Antibacterial Activity of PVA Hydrogels Embedding Oxide Nanostructures Sensitized by Noble Metals and Ruthenium Dye

Diana Pelinescu [1,†], Mihai Anastasescu [2,†], Veronica Bratan [2], Valentin-Adrian Maraloiu [3], Catalin Negrila [3], Daiana Mitrea [2], Jose Calderon-Moreno [2], Silviu Preda [2,*], Ioana Catalina Gîfu [4], Adrian Stan [5], Robertina Ionescu [1], Ileana Stoica [1], Crina Anastasescu [2,*], Maria Zaharescu [2] and Ioan Balint [2]

AFM characterization of TiO_2 and SiO_2 powders modified with Au andPt NPs

The morphology of the TiO_2 powder modified with AuNPs is presented in Figure S1a, while the TiO_2 powder modified with PtNPs ($PtTiO_2$ sample) is shown in Figure S1b. Each AFM image is accompanied by two arbitrary line-scans, plotted near the corresponding image. The Au and Pt morphologies, observed in Figures S1a,b, are replicated in the TiO_2 powders modified with AuNPs and PtNPs.

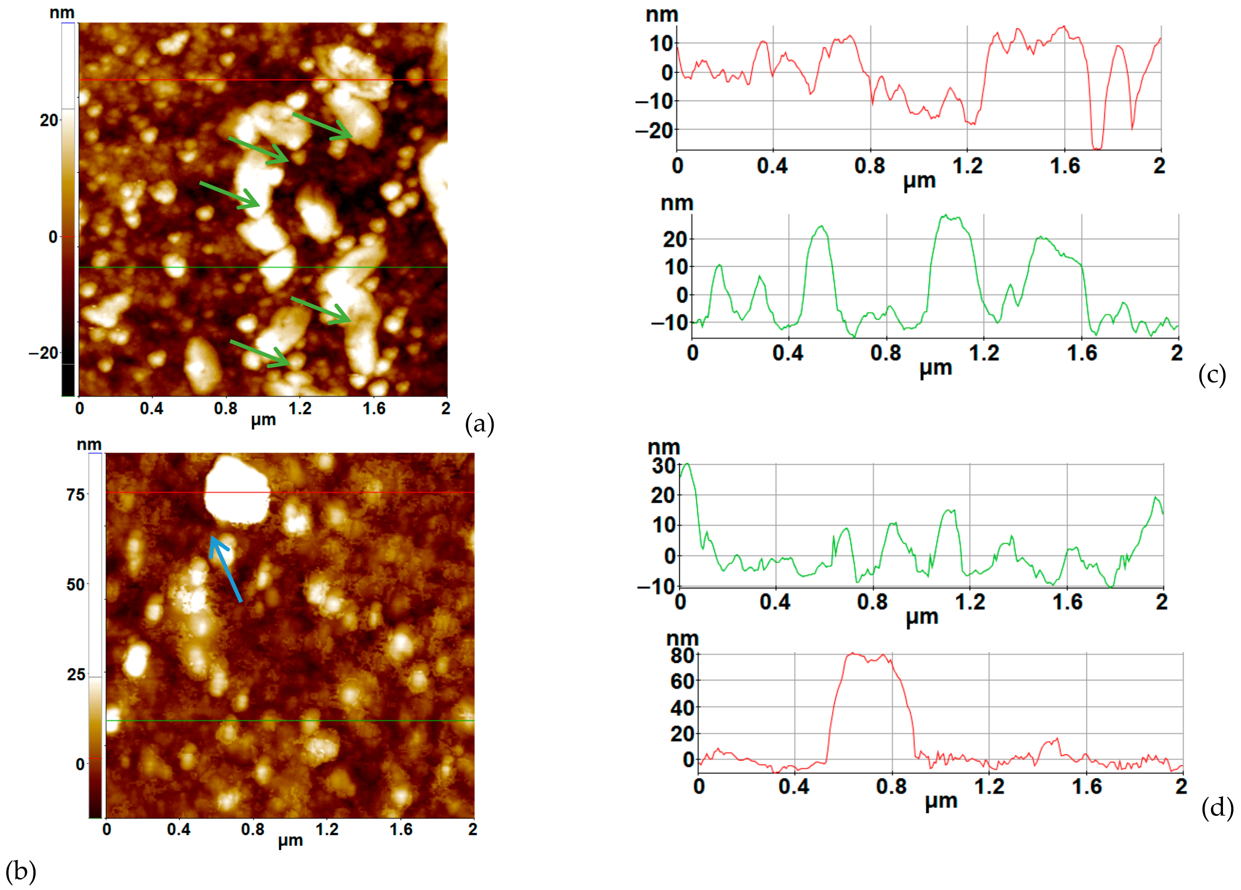

Figure S1. Topographic 2D AFM image of the TiO_2 powder modified with AuNPs (a) and, respectively, modified with PtNPs (b) scanned over (2μm x 2μm). Each AFM image is accompanied by two random line-scans (height *vs.* distance) corresponding to the scanned samples: TiO_2-AuNPs (c) and TiO_2-PtNPs (d).

The very small rounded particles (a few tens of nanometers in diameter) can be attributed to the TiO_2 matrix (anatase particles), while the larger ones, with a faceting

tendency can be assigned to AuNPs (indicated by the green arrows inserted for visual guidance in Figure S1a). In the Pt-modified TiO_2 material ($PtTiO_2$ - Figure S1b) a cluster of PtNPs (cauliflower-like) can be observed in the upper part (marked by the blue arrow). It can be also remarked that the morphology of Pt-modified TiO_2 ($PtTiO_2$) is slightly blurred, most likely due to the presence of sheet and nanowires, as indicated by TEM observations (Figure 2g from the Manuscript). The morphology of the SiO_2 powder modified with Au and Pt is presented in Figures S2 and S3.

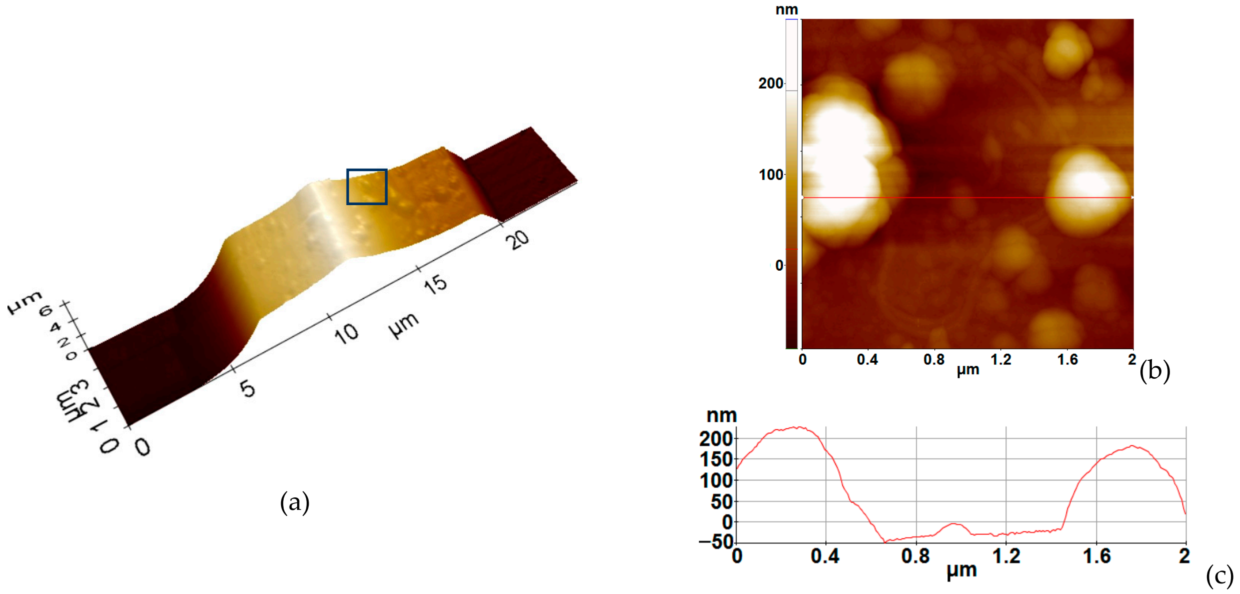

Figure S2. Part (slice) of a SiO_2 tube exhibited in a 3D topographic AFM image o at the scale of 4µm x 25µm – (a). Topographic 2D AFM image recorded on the walls of the SiO_2 tube modified with AuNPs scanned over (2µm x 2µm) – (b); plot of a line-scan (height *vs.* distance) no. 118 – (c).

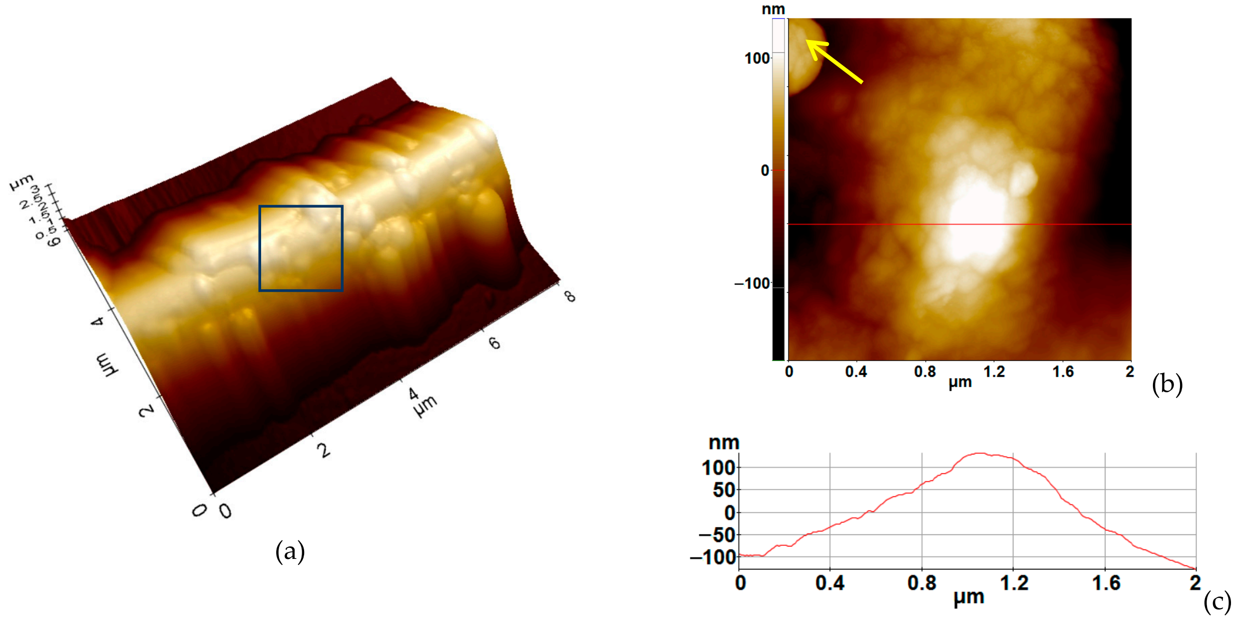

Figure S3. Part of a SiO_2 tube exhibited in a 3D topographic AFM image o at the scale of 6µm x 8µm – (a). Topographic 2D AFM image recorded on the walls of the SiO_2 tube modified with PtNPs scanned over (2µm x 2µm)– (b); plot of a line-scan (height vs. distance) – (c).

The morphology of the SiO_2 powders being predominant tubular [Refs. 26-28 from the Manuscript], the AFM images were collected by placing the AFM tip on-top of the wall of one of the SiO_2 tubes. Figure S2a shows a portion of a very large SiO_2 tube, modified with Au ($AuSiO_2$ sample), based on a 3D AFM image at the scale of 4µm x 25µm, exhibiting a distinct texture. Figure S2b was obtained by scanning an area of 2µm x 2µm on the tube-wall, as schematically marked by a square in Figure S2a. The tube-wall is not smooth but covered with small and large particles, from tens to hundreds of nm – as indicated by the line-scan from Figure S2c, attributable to spherical SiO_2 particles and AuNPs, as already observed in TEM (Figure 2a from the Manuscript). Figure S3a presents a part of another large SiO_2 tub, modified with Pt ($PtSiO_2$ sample) based on a 3D AFM image recorded at the scale of 6µm x 8µm. Further on, Figure S3b was recorded on the tube's wall, presenting its morphology in detail. The dispersion of PtNPs within the SiO_2 wall tube appears to be better (as indicated by the TEM image in Figure 2c from the Manuscript) than for the AuNPs (despite the presence of the cauliflower-like structures, as shown by the yellow arrow in the upper left corner of Figure S3b) and the line-scan profile in Figure S3c.

Hydroxyl radicals generation under simulated solar irradiation by SiO_2 and TiO_2 based powders, free and embedded in PVA gel:

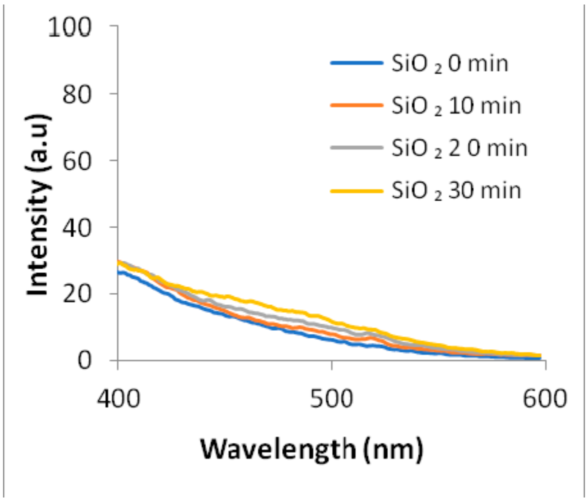

(a)

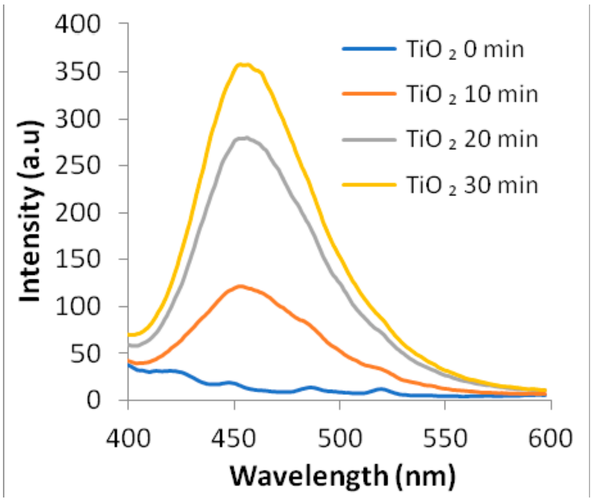

(j)

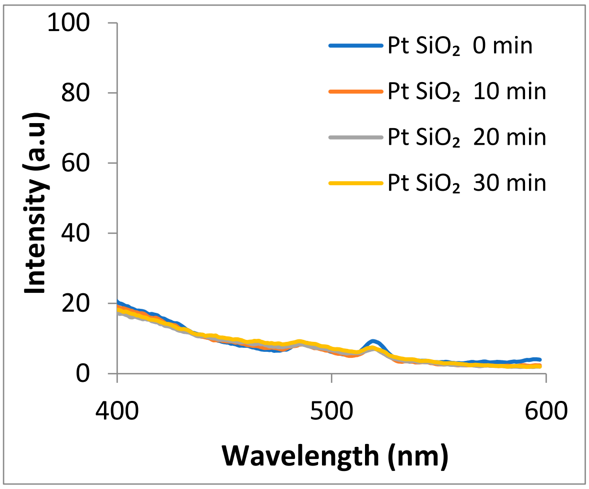

(b)

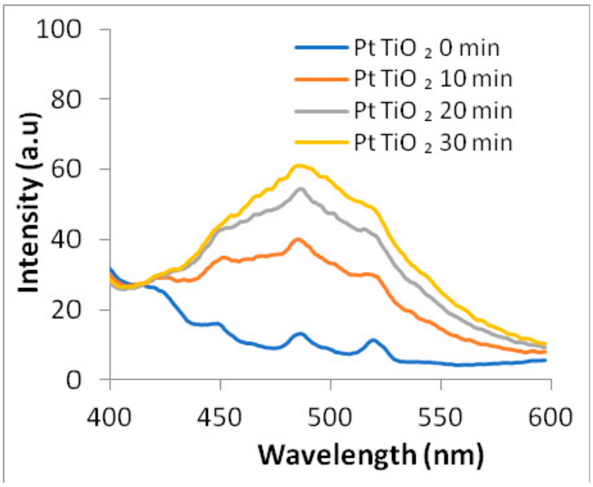

(k)

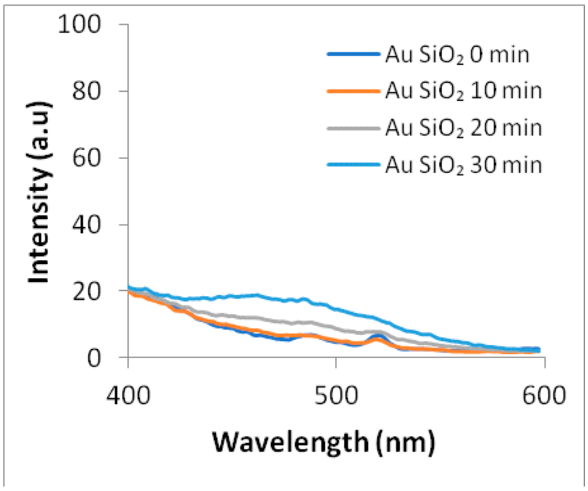
Au SiO_2 0 min
Au SiO_2 10 min
Au SiO_2 20 min
Au SiO_2 30 min
Intensity (a.u)
Wavelength (nm)
0
20
40
60
80
100
400
500
600

(c)

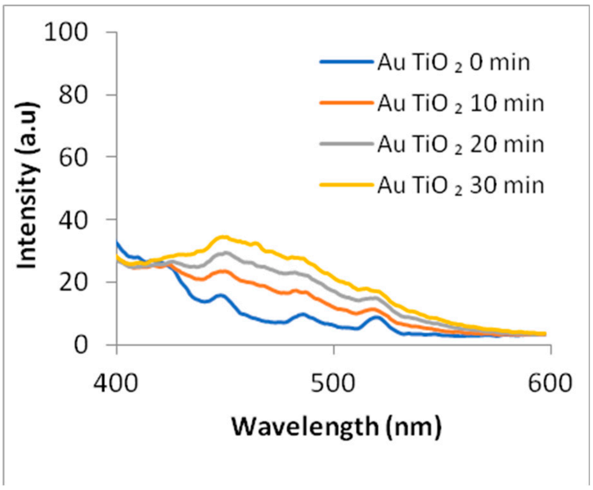
Au TiO_2 0 min
Au TiO_2 10 min
Au TiO_2 20 min
Au TiO_2 30 min
Intensity (a.u)
Wavelength (nm)
0
20
40
60
80
100
400
500
600

(l)

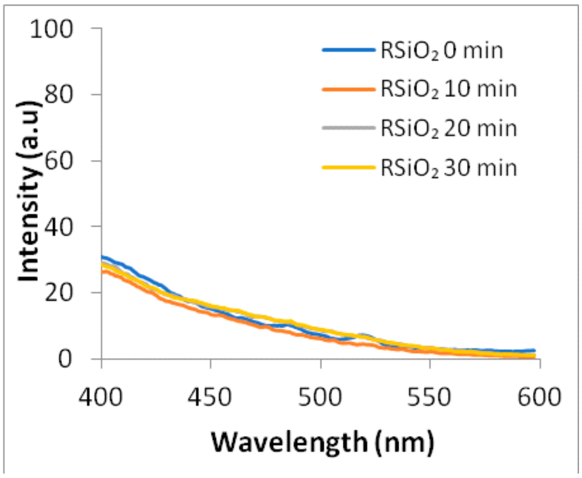
$RSiO_2$ 0 min
$RSiO_2$ 10 min
$RSiO_2$ 20 min
$RSiO_2$ 30 min
Intensity (a.u)
Wavelength (nm)
0
20
40
60
80
100
400
450
500
550
600

(d)

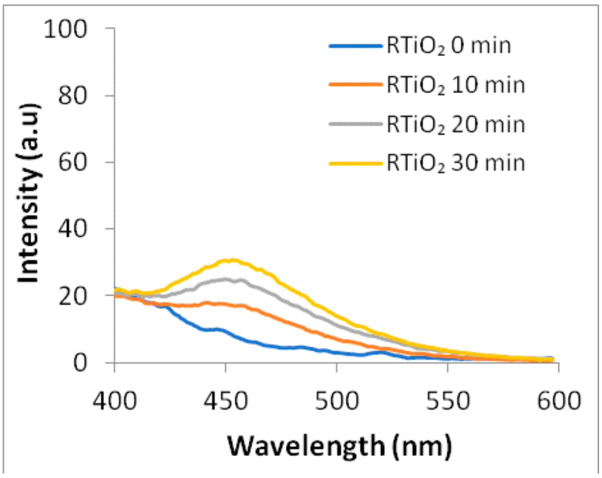
$RTiO_2$ 0 min
$RTiO_2$ 10 min
$RTiO_2$ 20 min
$RTiO_2$ 30 min
Intensity (a.u)
Wavelength (nm)
0
20
40
60
80
100
400
450
500
550
600

(m)

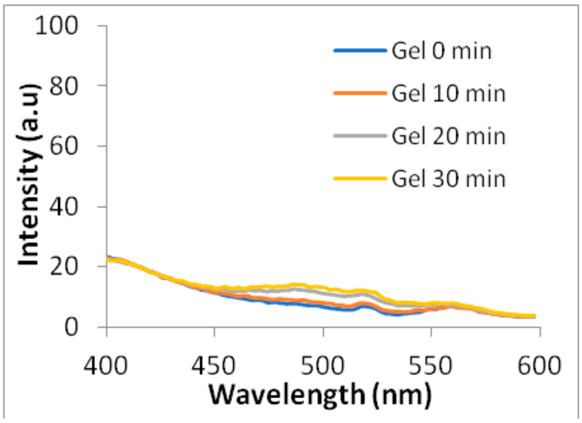
Gel 0 min
Gel 10 min
Gel 20 min
Gel 30 min
Intensity (a.u)
Wavelength (nm)
0
20
40
60
80
100
400
450
500
550
600

(e)

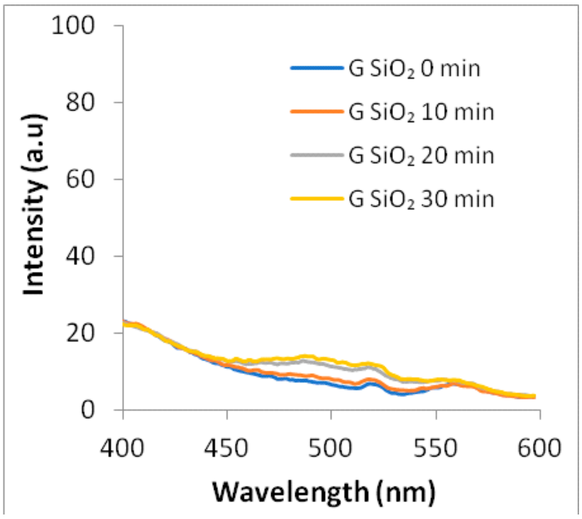
G SiO_2 0 min
G SiO_2 10 min
G SiO_2 20 min
G SiO_2 30 min
Intensity (a.u)
Wavelength (nm)

(f)

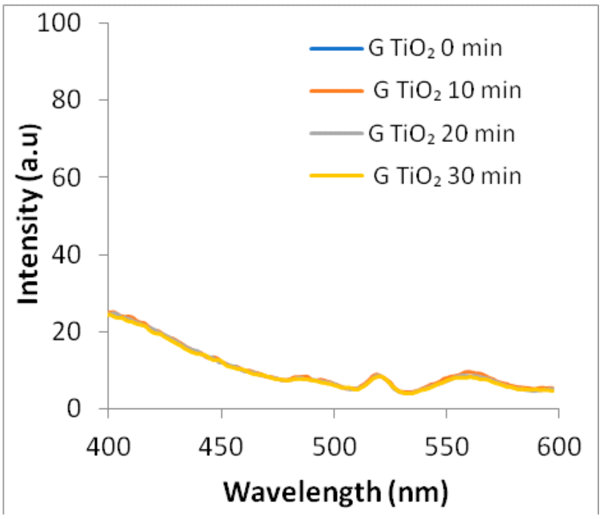
G TiO_2 0 min
G TiO_2 10 min
G TiO_2 20 min
G TiO_2 30 min
Intensity (a.u)
Wavelength (nm)

(n)

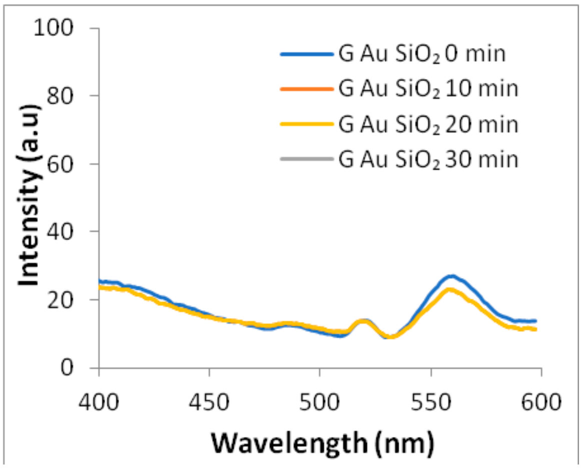
G Au SiO_2 0 min
G Au SiO_2 10 min
G Au SiO_2 20 min
G Au SiO_2 30 min
Intensity (a.u)
Wavelength (nm)

(g)

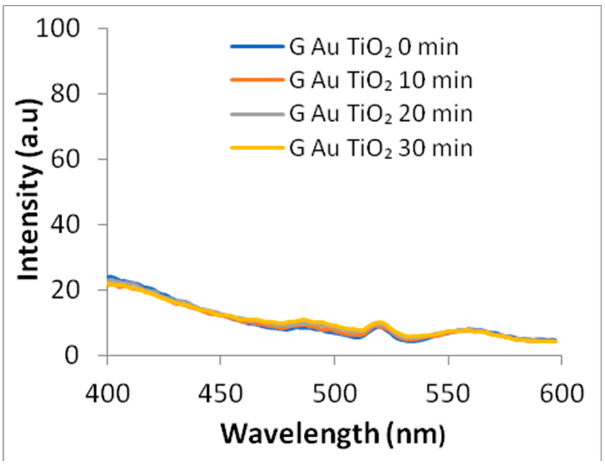
G Au TiO_2 0 min
G Au TiO_2 10 min
G Au TiO_2 20 min
G Au TiO_2 30 min
Intensity (a.u)
Wavelength (nm)

(o)

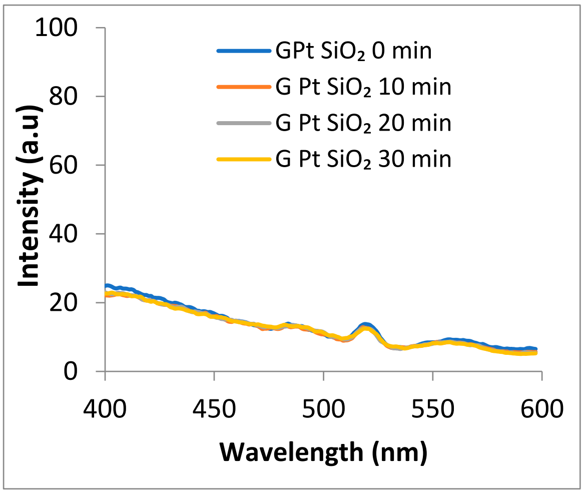
GPt SiO_2 0 min
G Pt SiO_2 10 min
G Pt SiO_2 20 min
G Pt SiO_2 30 min
Intensity (a.u)
Wavelength (nm)

(h)

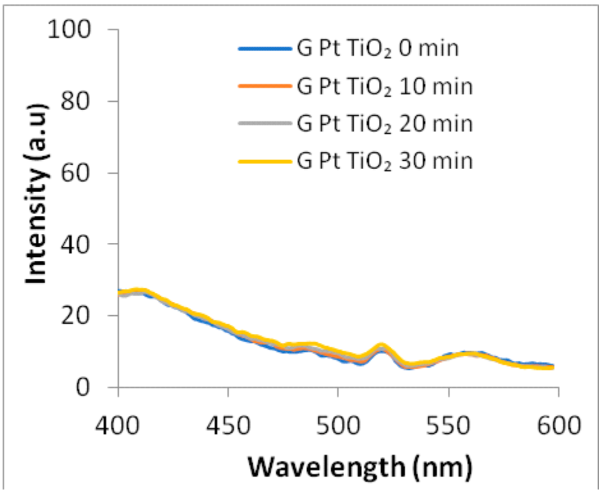
G Pt TiO_2 0 min
G Pt TiO_2 10 min
G Pt TiO_2 20 min
G Pt TiO_2 30 min
Intensity (a.u)
Wavelength (nm)

(p)

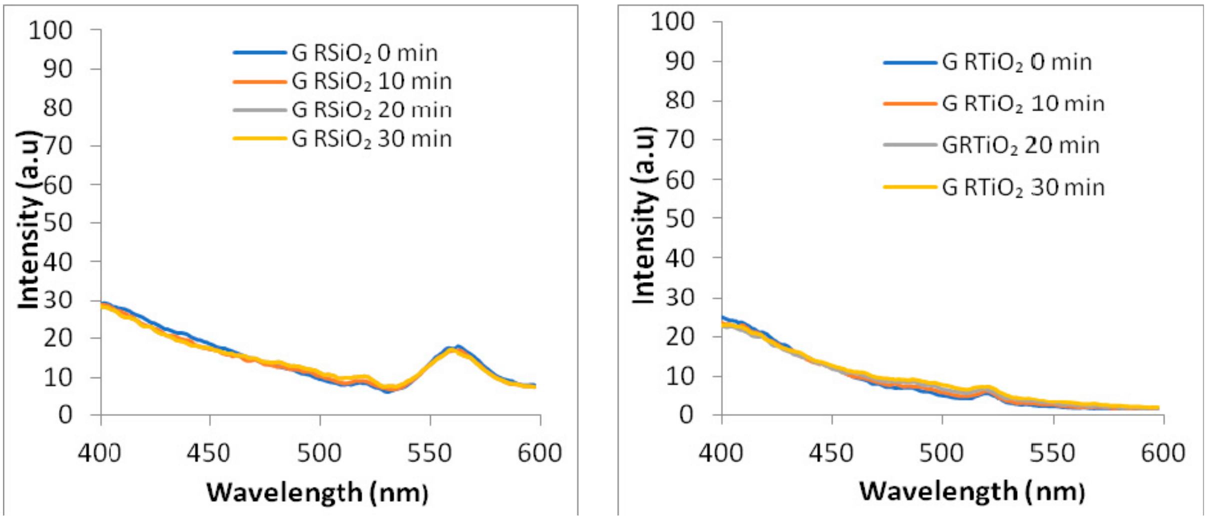

(i) (r)

Figure S4. The generation of the hydroxyl radicals under simulated solar irradiation by SiO_2 and TiO_2 based powders, free and embedded in PVA gel.

Electrokinetic Potential measurements

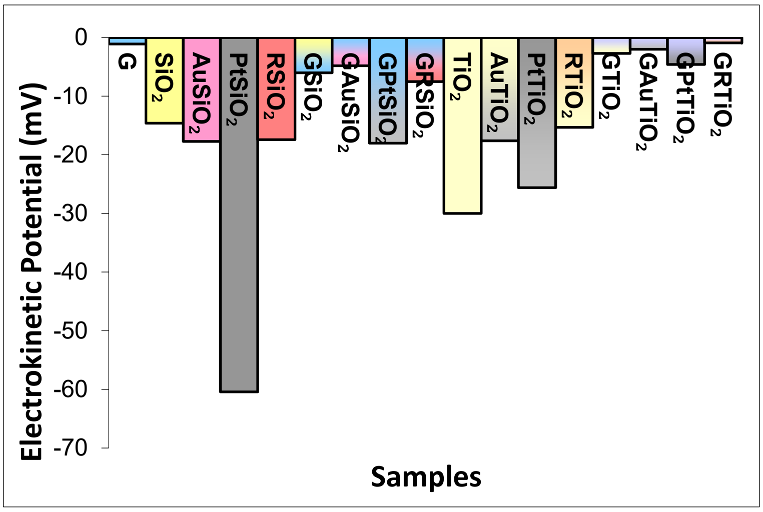

Figure S5. Electrokinetic potential measurements for SiO_2 and TiO_2-based powders free and embedded in PVA gel. The experiments were made in triplicates.
